# Depletion of HDAC1, 7 and 8 by Histone Deacetylase Inhibition Confers Elimination of Pancreatic Cancer Stem Cells in Combination with Gemcitabine

**DOI:** 10.1038/s41598-018-20004-0

**Published:** 2018-01-26

**Authors:** Mao-Hua Cai, Xiao-Gang Xu, Shi-Li Yan, Ze Sun, Yin Ying, Bai-Kui Wang, Yue-Xing Tu

**Affiliations:** 1 Department of General Surgery, Chun’an First People’s Hospital (Zhejiang Provincial People’s Hospital Chun’an Branch), Hangzhou, 311700 Zhejiang Province China; 2 Key Laboratory of Molecular Animal Nutrition of Ministry of Education, Institute of Feed Science, College of Animal Sciences, Zhejiang University, Hangzhou, 310029 Zhejiang Province China; 3 Zhejiang Academy of Traditional Chinese Medicine, Hangzhou, 310007 Zhejiang Province China; 4 Department of Critical Care Medicine, Zhejiang Provincial People’s Hospital, People’s Hospital of Hangzhou Medical College, Hangzhou, 310014 Zhejiang Province China

## Abstract

Trichostatin A (TSA) possess histone deacetylase (HDAC) inhibitory potential, can reverse the deactivation of tumor suppressor genes and inhibit tumor cell proliferation. We evaluated the effect of TSA on HDAC expression, tumor cell proliferation, and cancer stem cells (CSCs) activities in pancreatic ductal adenocarnoma (PDAC) cells. The PDAC cell lines MiaPaCa-2 and PANC-1 were distinctly sensitive to TSA, with enhanced apoptosis, compared to SAHA. TSA or SAHA inhibited vimentin, HDACs 1, 7 and 8, upregulated E-cadherin mRNA and protein levels in the PDAC cells, and time-dependently downregulated Oct-4, Sox-2, and Nanog, as well as inhibited PDAC tumorsphere formation. TSA also induces accumulation of acetylated histones, while increasing histone 3 lysine 4 or 9 dimethylation levels in PDAC cells and enhancing the epigenetic activity of SAHA. The anti-CSCs effect of TSA was like that obtained by silencing HDAC-1 or 7 using siRNA, and enhances Gemcitabine activity. Our study highlights the molecular targetability of HDACs 1, 7, and 8, confirm their PDAC-CSCs maintaining role, and demonstrate that compared to SAHA, TSA modulates the epigenetically- mediated oncogenic activity of PDAC-CSCs, and potentiate Gemcitabine therapeutic activity, making a case for further exploration of TSA activity alone or in combination with Gemcitabine in PDAC therapy.

## Introduction

Lately, the targetability of HDACs as an anticancer therapeutic strategy has received a lot of attention and become a subject of increased research activities^1^. Playing very vital roles in gene expression regulation, uncoiling of the closed ‘supercoiled’ chromatin, thus enhancing chromatin accessibility at promoter sites, and facilitating complex formation with DNA-binding proteins^2,3^. Currently, there are identified human HDACs grouped into four classes based on their yeast homology, subcellular localization, expression profile and substrate. Class 1 (HDACs 1, 2, 3, and 8), class 2 (HDACs 4–7, 9 and 10) and class 4 (HDAC 11) are Zn^2+^-dependent and utilize histones as substrates. The class 3 HDACs because of their similarity to yeast Sir2 protein, are referred to as sirtuins (SIRTs 1–7) and are NAD^+^ dependent^2^.

Because of the crucial role of HDACs as transcriptional co-repressors in many disease conditions, including cancer, and the effect of their mutation, aberrant expression or deregulation in cancer and other disorders, targeting and inhibiting HDAC activities is increasingly being demonstrated as effective anticancer therapy^4,5^. Presently, there are two United States Food and Drug Administration (FDA)-approved HDAC inhibitors for anticancer therapy, namely, Romodepsin (FK228) and Vironistat (otherwise, known as suberanilohydroxamic acid, SAHA), while a couple of others are undergoing clinical trial stage^6,7^. These HDAC inhibitors include the antibiotic apicidin^8^, fatty acid sodium butyrate^9^, and the cyclic peptide depsipeptide^10^, which has been shown to be class 1 HDAC- selective and alters histone modifications in cells at low nanomolar doses, as well as, the hydroxamic acids TSA^10^, which is class 1, 2 and 4 HDACs- selective and like depsipeptide, exhibit significant potency at very low dose.

All these compounds have been shown to possess promising anticancer potential, however, unraveling the underlying mechanism by which their HDAC inhibitory activities induce or facilitate inhibition of cancer growth remains unclear, especially in the light of the differential expression of various HDACs in different tumors, non-specific therapeutic effect and the differential response of cancer cells to HDAC inhibitors^10,11^.

In this present study, we accessed and assessed the the GEO dataset GSE2873, analyzed the differential expression profile of class 1, 2 and 4 HDACs in paired pancreatic duactal adenocarcinoma (PDAC) and adjacent non-tumor tissue samples, identified and delineated three HDACs, namely HDACs 1, 7 and 8, with PDAC - specific statistically relevant overexpression. We also evaluated the diagnostic and prognostic relevant of these HDACs in PDAC patients, as well as characterized the responsiveness of PDAC cell lines PANC-1 and MiaPaCa-2 to HDAC inhibitors TSA, SAHA, and/or chemotherapeutic agent Gemcitabine. Here, we assessed the HDAC inhibitory activity, anticancer efficacy and selectivity of these compounds by evaluating their effects on the PANC-1 and MiaPaCa-2 cells, as well as sought to understand the relationship between their observed ability to inhibit HDAC expression and/or activity and their impact on PDAC cell viability and oncogenicity. Furthermore, we identified disease-relevant drug-induced and HDAC-mediated marked loss of CSCs phenotypes increased chemosensitivity. This finding broadens our understanding of HDAC-mediated anticancer activity, and further makes the case for continued research into the therapeutic effect of HDAC inhibitors, as well as their potential use in personalized medicine applications.

## Results

### The preferential overexpression of HDACs 1, 7 or 8 in PDAC samples has prognostic relevance for pancreatic cancer patients

**Figure 1:**
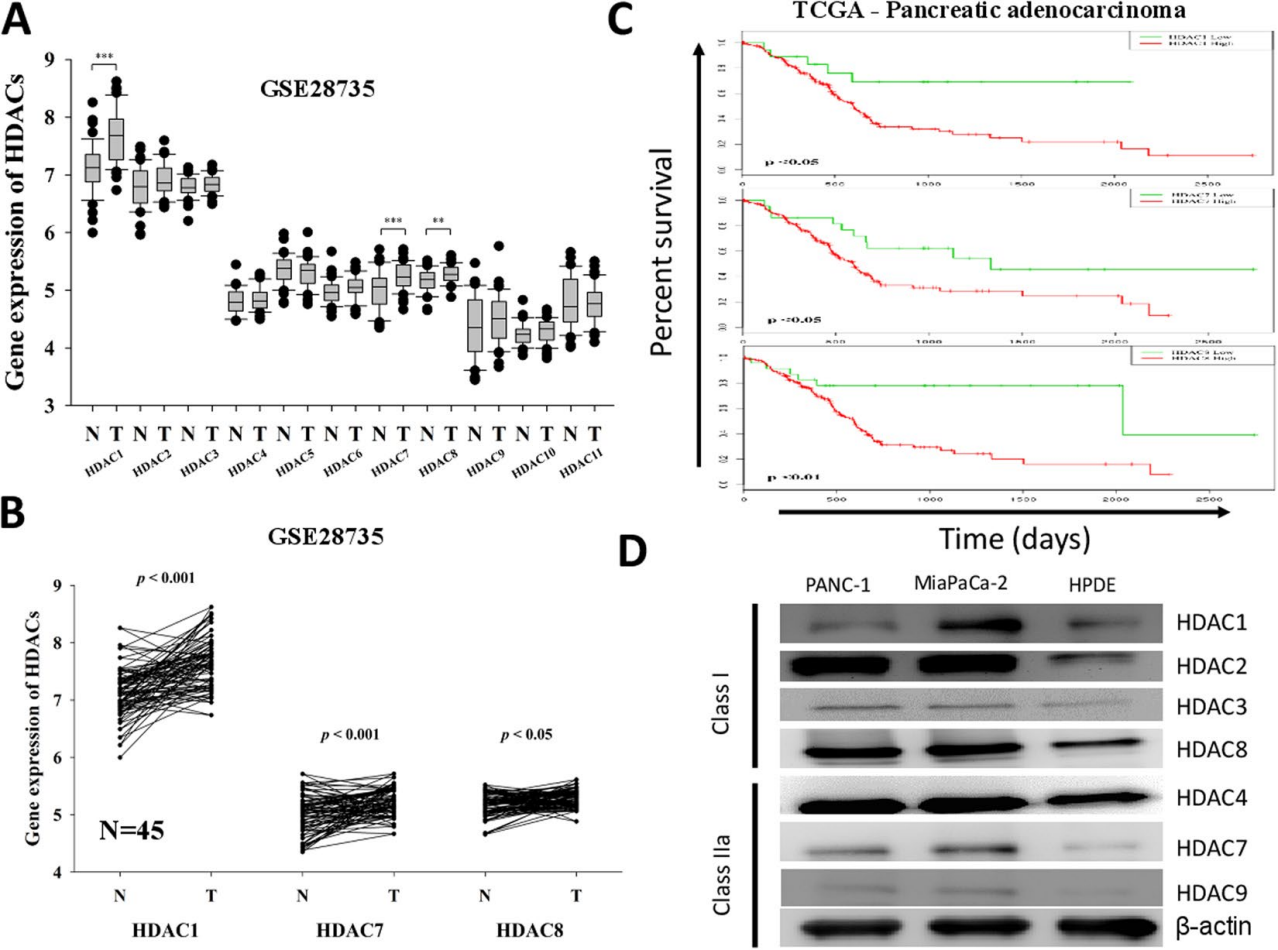
The preferential overexpression of HDACs 1, 7 or 8 in PDAC samples has prognostic relevance for pancreatic cancer patients. (**A**) Relative mRNA expression of class 1, class 2 and class 4 HDACs in pancreatic adenocarcinoma (T) and adjacent normal (N) tissues in GSE28735 dataset, n = 45. (**B**) Differential expression of HDAC1, 7, and 8 in the 45 paired PDAC and normal tissues. (**C**) Kaplan-Meier analysis of HDACs gene expression in TCGA PAAD cohort. Patients with low HDAC1 (n = 18), 7 (n = 22) and 8 (n = 23) expression had longer overall survival (OS) than those with high HDAC1 (n = 158), 7 (n = 154), and 8 (n = 153) expression. *p < 0.05, **p < 0.01, ***p < 0.001. (**D**) Expression of classes I and II HDACs was determined by Western blotting in PANC-1, MIAPaCa-2 and HPDE cell line.

To understand the role of HDACs in PDAC, we analyzed the differential expression profile of class 1, 2 and 4 HDACs in paired pancreatic duactal adenocarcinoma (PDAC) and adjacent non-tumor tissue samples based on the GSE2873 data accessed on the GEO. Analysis of this dataset (n = 45) revealed that HDAC 1, 7 and 8 were overexpressed in the tumor (T) samples compared to the non-tumor (NT) groups, and that only the differential expression profile of HDACs 1, 7 and 8 were statistically very significant, compared with the other HDACs (Fig. 1A), with p < 0.001, p < 0.001 and p < 0.05, respectively (Fig. 1B). We then evaluated if these HDACs had any prognostic relevance by accessing and analyzing The Cancer Genome Atlas (TGCA) pancreatic adenocarcinoma (PAAD) dataset (n = 183). Using the Kaplan-Meier curve, we demonstrated that PDAC patients with high HDAC 1, 7 or 8 expressions had significantly worse overall survival (p < 0.05, p < 0.05, and p < 0.01, respectively), compared to those with low HDACs 1, 7 or 8 expression levels (Fig. 1C). Expression of classes I and II HDACs was determined by Western blotting in HPDE, PANC-1 and MIAPaCa-2 cell line. The class I HDACs (1, 2, 3, and 8) were detected in both cell lines though the levels were variable. In general, the levels of the class I HDACs in the HPDE cells were relatively lower compared to the majority of the PANC-1 and MIAPaCa-2 cell lines. Interestingly, most of class IIa HDACs were detected in both in PANC-1 and MIAPaCa-2 cells but not in the HPDE cells (Fig. 1D). These data do suggest that the preferential overexpression of HDAC1, 7 or 8 in PDAC samples is prognostically-relevant for pancreatic cancer patients.

### Trichostatin A and vorinostat suppress the viability of human PDAC cells

**Figure 2:**
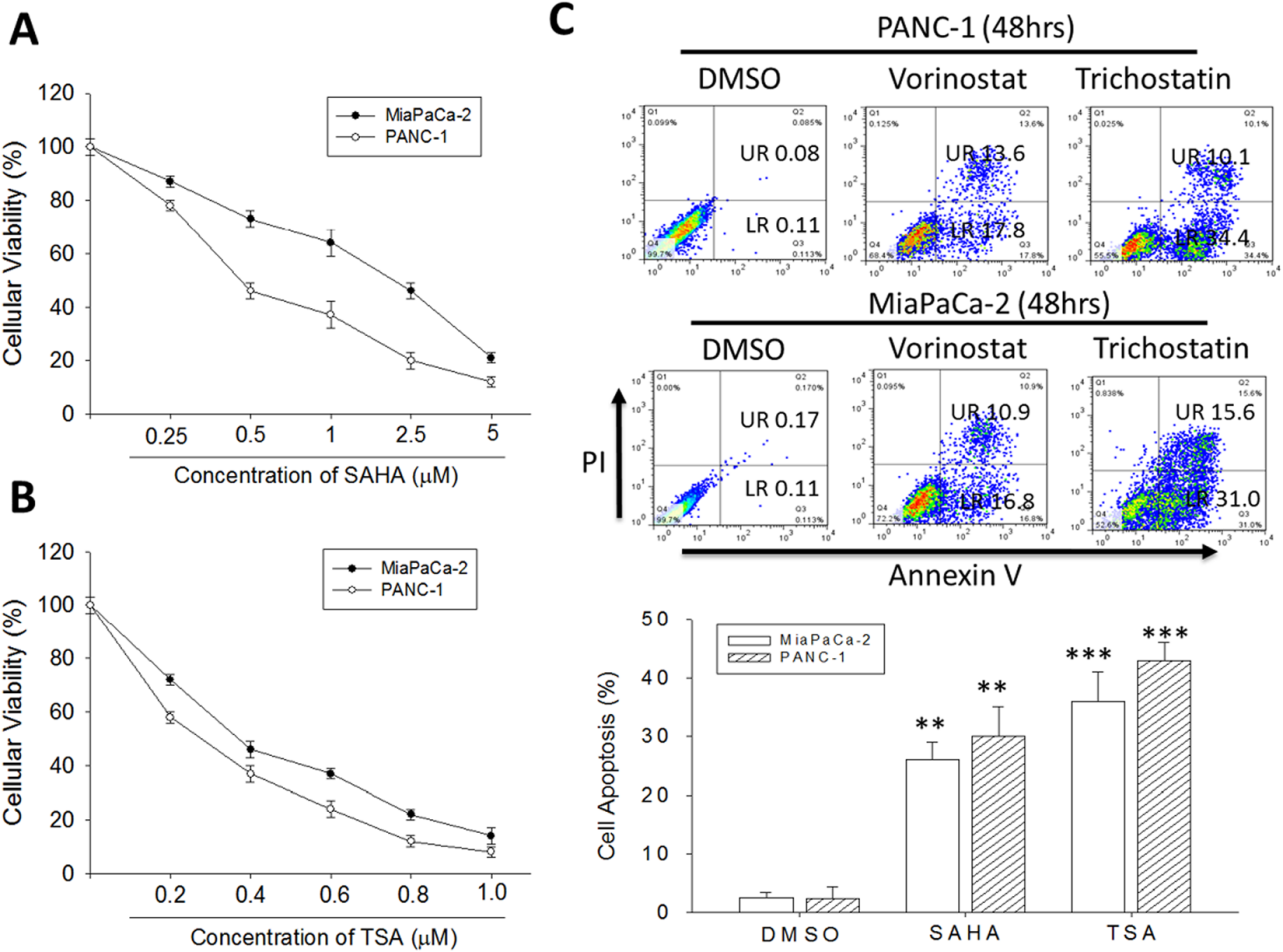
Trichostatin A and Vorinostat suppress the viability of human PDAC cells. Line chart of the cellular viability of (**A**) SAHA-treated or (**B**) TSA-treated PANC-1 and MiaPaCa-2 cells. (**C**) The percentages of Annexin V/PI-labeled cells in PANC-1 and MiaPaCa-2 cells, after SAHA or TSA treatment, compared with DMSO-treated control cells, analyzed by flow cytometry (upper panel). Graph showing the percentage of apoptotic cells (lower panel). UR, upper right. LR, lower right. SAHA, Vorinostat. SA, Trichostatin. *p < 0.05, **p < 0.01, ***p < 0.001.

To assess the effect of TSA and SAHA on human PDAC cells, we treated MiaPaCa-2 or PANC-1 cells with 0.25–5 µM SAHA or 0.2–1 µM TSA for 48 h and then evaluated their survival using the SRB assay. Our results showed that TSA or SAHA dose-dependently suppressed the viability of the MiaPaCa-2 and PANC-1 cell lines. The IC_50_ of SAHA in MiaPaCa-2 or PANC-1 cells was 1.83 ± 0.52 or 0.47 ± 0.21 µM, respectively (Fig. 2A), and the IC_50_ for TSA was much lower, with 0.38 ± 0.15 or 0.29 ± 0.04 µM, MiaPaCa-2 or PANC-1 cells, respectively (Fig. 2B). We extended our studies to a time-dependent curve of IC_50_ value by SAHA and TSA. SAHA and TSA significantly decreased survival of MiaPaCa-2 or PANC-1 in a time-dependent manner. In most cell lines, IC_50_ values were 2–3 times lower for TSA than SAHA (Supplementary Fig. S1). In similar experiments to assess the therapeutic effect of TSA and SAHA, using the Annexin V/PI assay, we observed significant induction of cell death in PANC-1 and MiaPaCa-2 cells treated with 1 M TSA or SAHA for 48 h, compared to the DMSO- treated control cells. This tumor-killing effect was more apparent in the cells treated with 1 µM TSA (Fig. 2C). As demonstrated, compared to the control cells treated with DMSO, treatment with 1 M SAHA induced 12-fold and 10.8-fold more apoptotic (late and early) PANC-1 and MiaPaCa-2 cells, respectively, while TSA treatment of the PANC-1 and MiaPaCa-2 cells caused 17.2-fold and 15-fold more cell deaths. These findings indicate the cytotoxic effect of TSA and SAHA in both primary PANC-1 and metastatic MiaPaCa-2 of human PDAC cell lines, and confirm the crucial role of apoptosis in TSA or SAHA anticancer activity, and the sensitivity of the cancer cells to a low-dose regimen.

### Trichostatin A and Vorinostat suppresses HDAC 1, 7 or 8, as well as Vimentin, while upregulating E-cadherin expression levels in PDAC cells, at mRNA and protein levels

**Figure 3:**
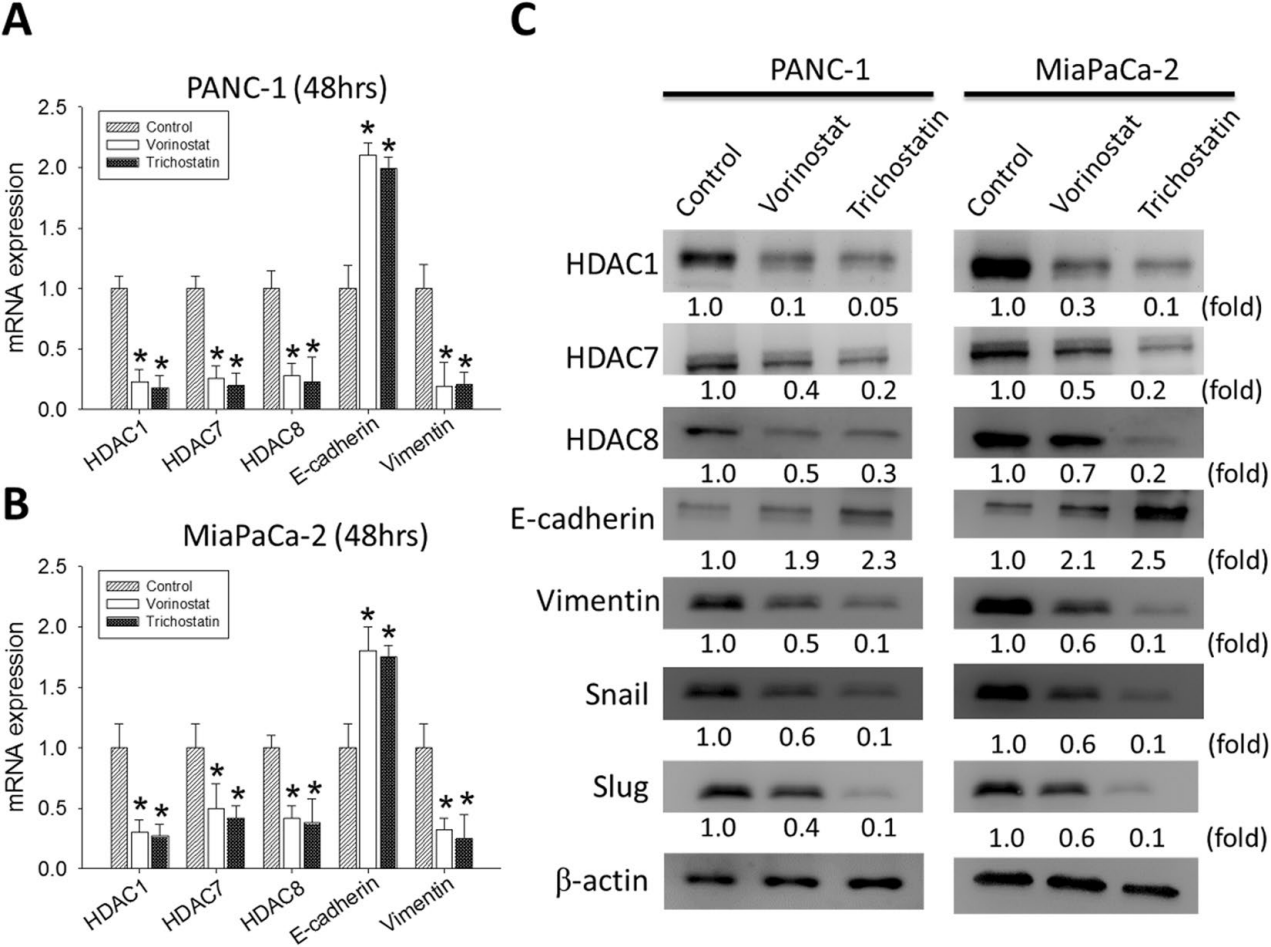
Trichostatin A and Vorinostat significantly suppressed the expression levels of HDAC1, 7 and 8, as well as vimentin, while upregulating E-cadherin expression in PANC-1 and MiaPaCa-2, at mRNA and protein levels. Graph of the effect of SAHA or TSA on the mRNA expression of HDAC1, HDAC7, HDAC8, E-cadherin and vimentin in (**A**) PANC-1 and (**B**) MiaPaCa-2 cells, compared with the untreated control cells. GAPDH mRNA was used as the control. (**C**) Changes in HDAC1, HDAC7, HDAC8, E-cadherin, vimentin, slug and snail protein expression after SAHA or TSA treatment. Fold change in expression is indicated. β-actin was loading control. *p < 0.05, **p < 0.01, ***p < 0.001.

Having shown that HDACs 1, 7 and 8 are relevant in the pathogenesis and prognosis of PDAC, as well as cytotoxic effects of TSA and SAHA in PDAC cells, we then investigate the effect of TSA or SAHA on the HDAC- rich and -enabled PDAC cells. Our results demonstrate that 48 h treatment of the PANC-1 cells with 1 µM SAHA or TSA significantly inhibited the mRNA expression of HDAC1, HDAC7, HDAC8 and Vimentin, while concurrently upregulating E-cadherin expression, compared to the untreated control cells (Fig. 3A). Comparable results were obtained after treatment of the MiaPaCa-2 cells with SAHA or TSA for 48 hours (Fig. 3B). In parallel experiments using the Western blot assay, we demonstrated that the observed inhibitory effect of SAHA or TSA on the PDAC cells was not mRNA-specific, but was reproducible on the protein level. SAHA or TSA treatment significantly inhibited the expression of HDAC1, HDAC7, HDAC8 and Vimentin protein, but enhanced E-cadherin expression. To confirm possible anti-EMT activity, SAHA or TSA -treated cells were examined for the EMT marker snail and slug. Expression of snail and slug were indeed decreased by dose-dependent SAHA or TSA (Fig. 3C). We next investigated the effect of SAHS and TSA on growth and survival of pancreatic cancer cells. Treatment of MiaPaCa-2 and PANC-1 with SAHA (0, 0.5, and 1 μM) or TSA (0, 0.4, and 0.6 μM) for 48hrs potently suppressed cellular proliferation as quantified by bromodeoxyuridine (BrdU) incorporation assay (Supplementary Fig. S2). These results are suggestive of a probable role of dysregulated HDACs 1, 7 and 8 expressions in epithelial-to-mesenchymal transition (EMT) and PDAC cell survival.

### Trichostatin A and Vorinostat epigenetically regulate the CSCs activities in PDAC cells by modulating the expression of CSC markers Oct-4, SOX-2, and Nanog, time dependently

**Figure 4:**
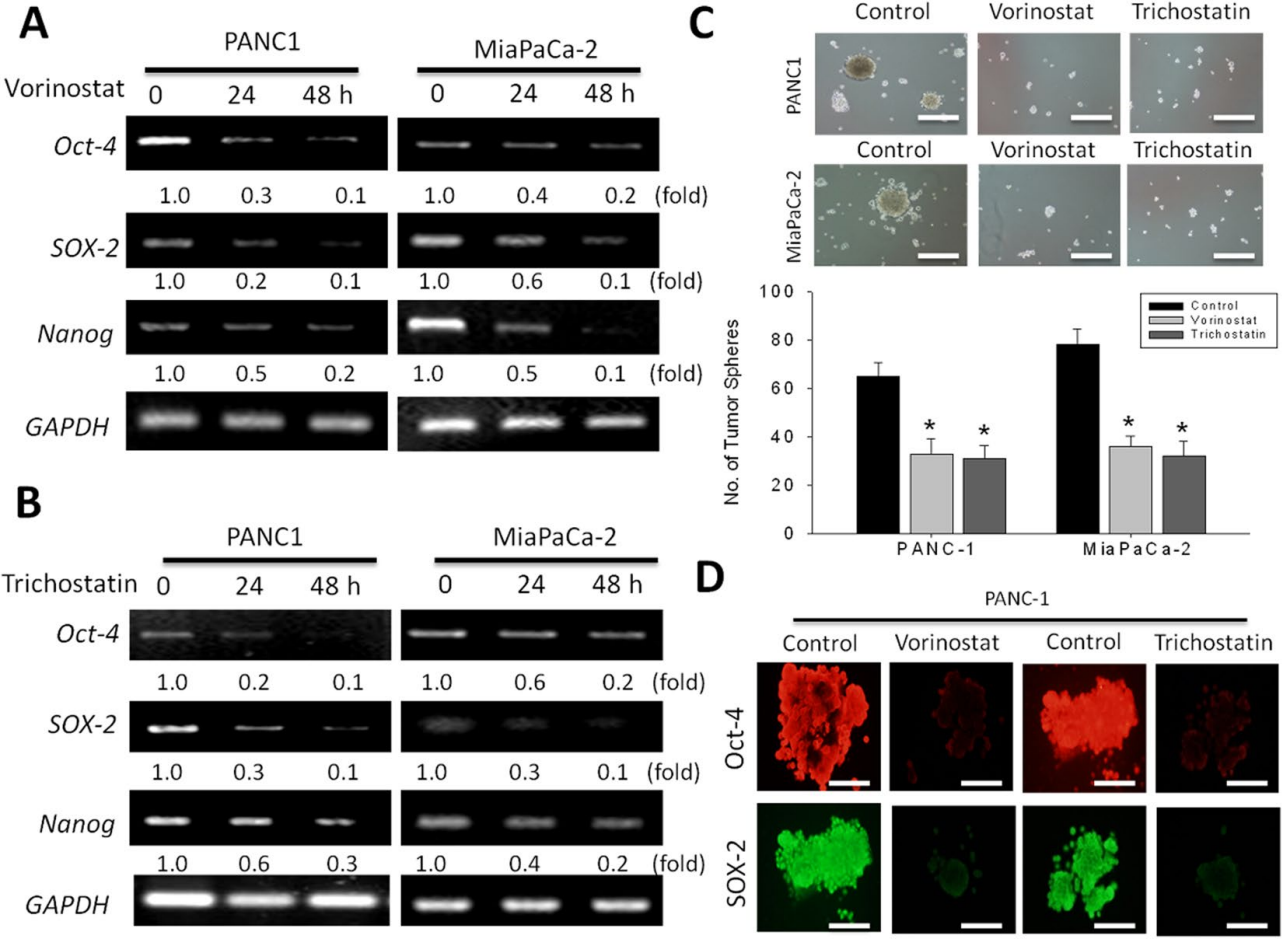
Trichostatin A and Vorinostat epigenetically regulate the CSC activities in PDAC cells by modulating the expression of CSC markers Oct-4, SOX-2, and Nanog, time dependently. The effect of (**A**) SAHA and (**B**) TSA treatement on the mRNA expression of Oct-4, Sox-2, and Nanog in PANC-1 or MiaPaCa-2 cells at 24 h and 48 h time-point. GAPDH was used as internal control. The relative expression of mRNA is shown as the fold ratio of the density of each sample to the GAPDH density of RT-PCR products. (**C**) Images of tumor sphere formation in PANC-1 and MiaPaCa-2 cells pre-treated with SAHA or TSA. *p < 0.05, **p < 0.01, ***p < 0.001. (**D**) SAHA and TSA suppresses sphere-forming PANC-1 cells expressing CSC markers. Immunocytochemical analyses of Oct-4 and SOX-2 in sphere-forming PANC-1 cells after treatment with SAHA and TSA for 5 days.

Since the presence of CSCs have been demonstrated in pancreatic cancer and implicated in the aggressive phenotype of PDAC cells^12–14^, we evaluated if SAHA or TSA exhibits any inhibitory effect on the CSCs- like phenotype of PDAC cells and their associated aggressiveness, by examining the effect of 1 M SAHA or TSA treatment on the mRNA expression of CSC transcription factors - Oct-4, Sox-2 and Nanog in the PANC-1 or MiaPaca-2 at the 24 h and 48 h time points. Results of our RT-PCR demonstrate that SAHA (Fig. 4A) and TSA (Fig. 4B) induce a time-dependent downregulation of Oct-4, Sox-2 and Nanog in both PDAC cell lines, compared to the untreated cells. Consistent with these data, our tumor sphere formation assay revealed significantly reduced ability of the PANC-1 and MiaPaCa-2 cells to form tumor spheres, when pre-treated with 1 µM SAHA or TSA. After SAHA or TSA treatment, a 1.88-fold (p < 0.05) and 2.00-fold (p < 0.05) reduction in the number of tumor spheres formed was observed in the PANC-1 cells, while for the MiaPaCA-2 cells, a 2.17-fold (p < 0.05) and 2.44-fold (p < 0.05) decrease in tumor sphere formation potential was noted after SAHA or TSA treatment, respectively (Fig. 4C). Immunocyto-chemical analyses showed that treatment with SAHA or TSA caused down-regulation of not only OCT-4 but also other stem cell markers, namely, SOX-2 (Fig. 4D). This data suggests an association between the observed PDAC cell death formation, reduced expression of HDACs 1, 7 and 8, downregulation of Oct-4, Sox-2 and Nanog, and loss of tumor sphere formation efficiency.

### Trichostatin A induces the accumulation of acetylated histones H3 and H4 in PDAC cells and enhances the epigenetic activity of Vorinostat

**Figure 5:**
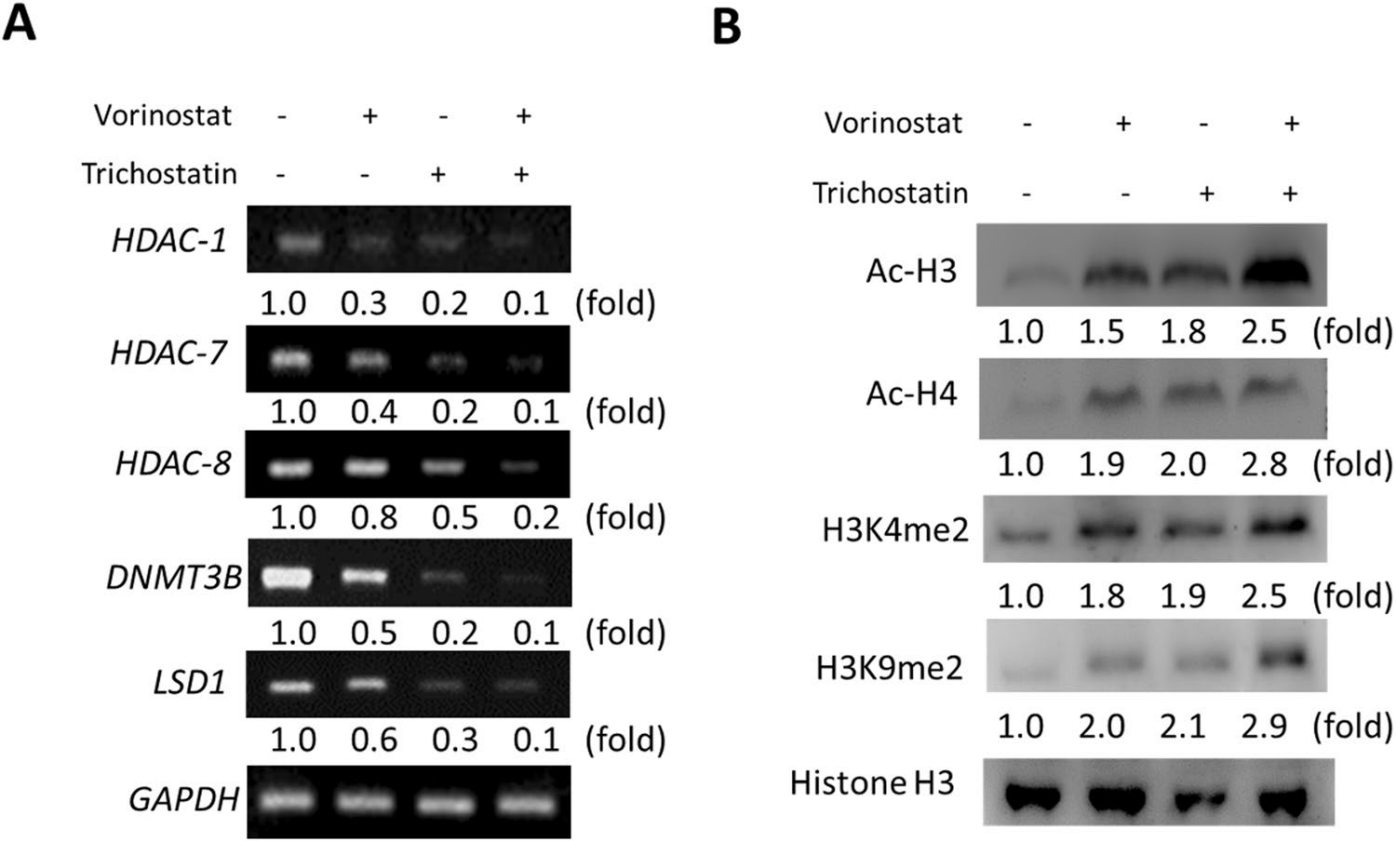
Trichostatin A induces the accumulation of acetylated histones H3 and H4 in PDAC cells and enhances the epigenetic activity of Vorinostat. (**A**) RT-PCR data showing co-reduction in the mRNA expression of HDAC-1, HDAC-7, HDAC-8, DNMT3B and LSD1 after treatemnt with SAHA and/or TSA. GAPDH mRNA was used as internal control. (**B**) Changes in the protein expression of Ac-H3, Ac-H4, H3K4me2, and H3K9me2 after treatment with SAHA and/or TSA. Histone H3 was used as loading control. Fold changes is indicated.

Understanding that the potent HDAC inhibitory effect of SAHA is associated with the accumulation of acetylated histones and non-histone proteins^15^, and having observed a probable association between downregulation of HDACs 1, 7, and 8 expression levels, TSA-induced PDAC apoptosis, reduced expression of pluripotent transcription factors and loss of tumor sphere formation efficiency, we sought to validate the relationship between TSA HDAC inhibitory effect and its anticancer activity. Thus, we probed the effect of TSA on HDACs 1, 7, and 8, the oncogenic *de novo* methyltransferase DNMT3B which abnormally methylates tumor suppressor genes^16^, and LSD1, which has been shown to sustain pancreatic cancer growth^17^. Our result showed co-downregulation of HDAC1, HDAC7, HDAC8, DNMT3B and LSD1 mRNA expression by 1 µM SAHA or TSA, however, this was more significant and apparent with TSA treatment. We also demonstrated by using concomitant administration of SAHA and TSA, that TSA enhances the therapeutic effect of SAHA (Fig. 5A). Furthermore, our Western blot analysis revealed that TSA significantly upregulate the expression of Ac-H3, Ac-H4, H3K4me2 and H3K9me2 proteins, as well as enhance the ability of SAHA to do same (Fig. 5B). These data highlight a probable direct connection between TSA HDAC inhibitory effect and its anticancer activity.

### TSA enhances the sensitivity of PDAC cells to Gemcitabine and increases its CSCs- targeting potential

**Figure 6:**
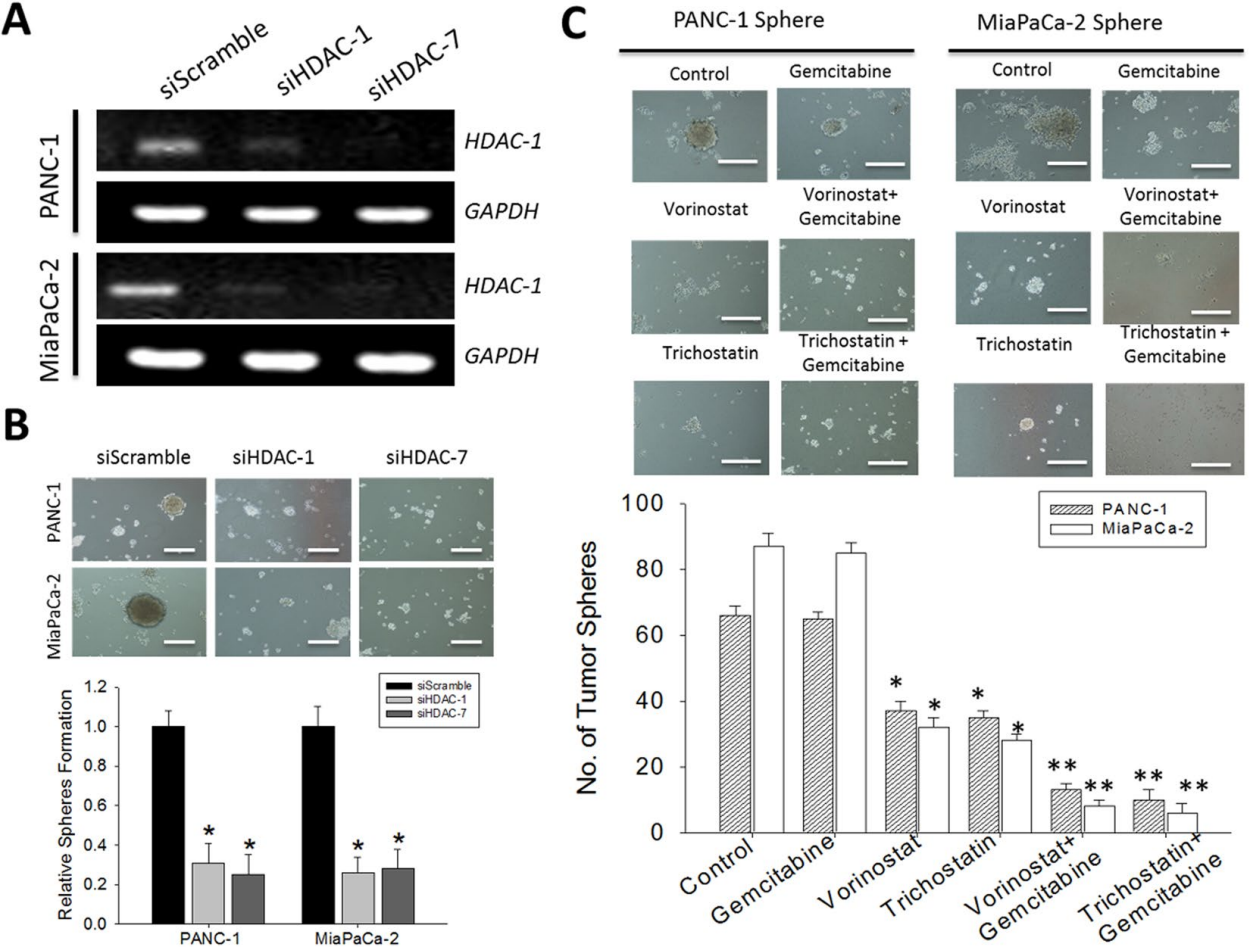
The anti-CSCs activity of Gemcitabine is enhanced in the presence of Trichostatin A or Vorinostat. (**A**) Knockdown efficiency of siHDAC-1 and siHDAC-7 in PANC-1 and MiaPaCa-2 cells (**B**) Images of sphere formation in siHDAC-1 or siHDAC-7 -infected PANC-1 and MiaPaCa-2, compared with scramble infected control cells. (**C**) Images showing the effect of Gemcitabine treatment of PANC-1 and MiaPaCa-2 cell-generated tumor spheres in the presence or absence of SAHA or TSA.

Since Gemcitabine is the current first line therapy for pancreatic cancer patients, we investigated the effect of TSA on the activity of standard chemotherapeutic agent. We treated PANC-1 and MiaPaCa-2 cells with Gemcitabine in the presence or absence of TSA or SAHA, and examined the evaluated its effect on tumor sphere formation while concurrently assessing for similarity with silencing HDAC 1 or 7 expression. After confirming the knockdown efficiency of our short interfering RNA-mediated HDAC-1 and HDAC-7 silencing in the PANC-1 and MiaPaCa-2 cells (Fig. 6A), consistent with data presented above, we demonstrated that the siHDAC-1 and siHDAC-7 infected PDAC cells significantly lost their ability to formed tumor spheres (Fig. 6B). Furthermore, no effect on cell proliferation or apoptosis were observed. We determined whether or not the expression of HDAC contributes to the EMT of pancreatic cancer cells. To test this hypothesis, we examined the expression levels of epithelial and mesenchymal markers. The upregulation of E-cadherin and the downregulation of vimentin were detected at mRNA in PANC-1 and MiaPaCa-2 cells. These results indicated that HDAC is important for PANC-1 and MiaPaCa-2 cells to maintain mesenchymal characteristics (Supplementary Fig. S3). In similar experiments using PDAC-CSCs spheres, we showed that while Gemcitabine mildly reduces the tumor sphere formation potential of the PDAC cells, combining Gemcitabine with SAHA or TSA enhances this effect, however, this enhanced tumor sphere inhibitory activity was observed in the TSA-Gemcitabine group (Fig. 6C).

### TSA treatment significantly suppressed tumor initiating ability *in vivo*

**Figure 7:**
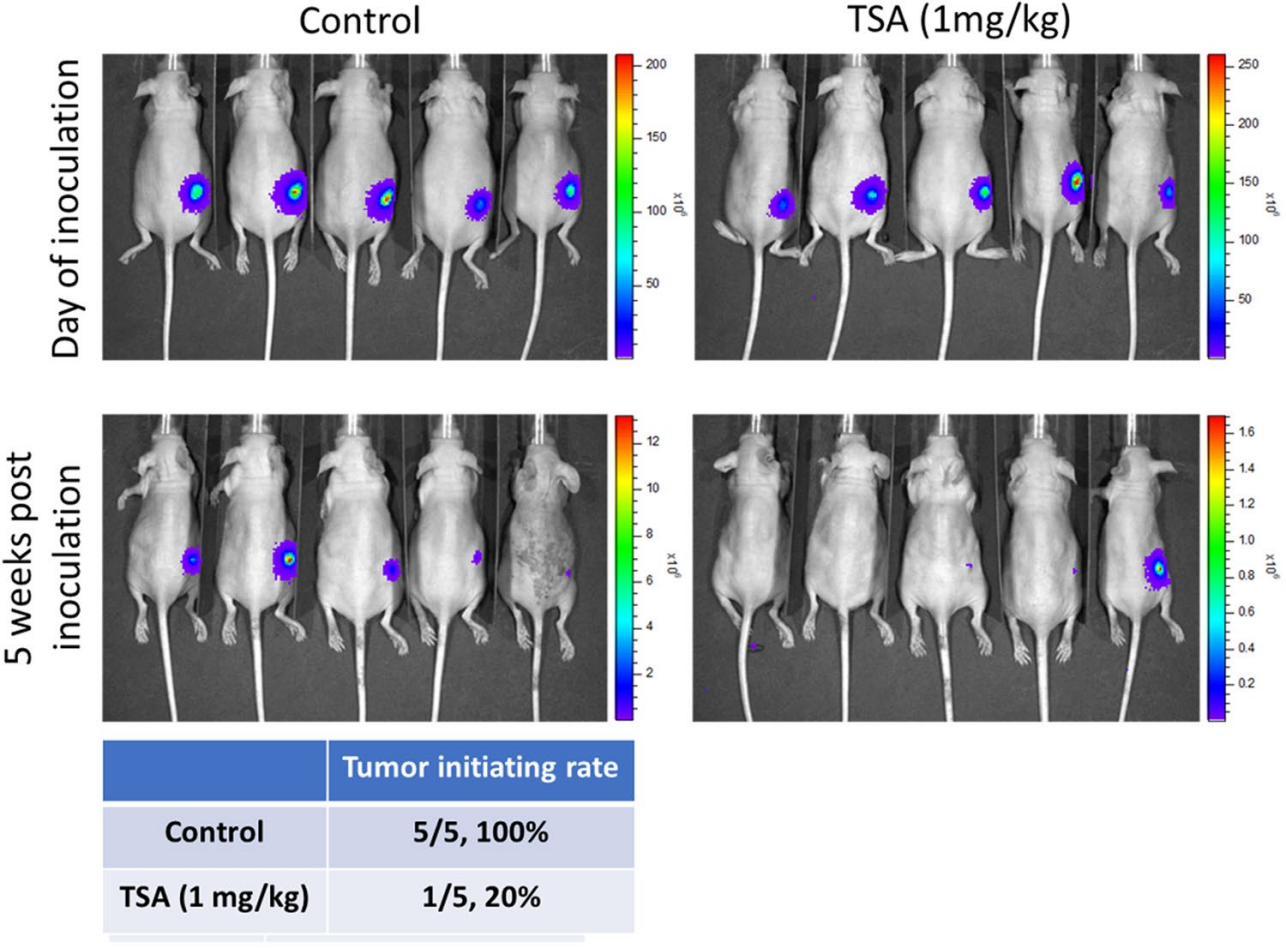
Trichostatin A (1 mg/kg, i.p injection, 3 times/week) suppressed tumor initiation by PANC-1 tumor spheres. PANC-1 tumor spheres (5,000 cells/injection) were subcutaneously injected into the flanks of NOD/SCID mice to establish xenograft model. Bioluminescence was used to track the tumor initiation of PANC-1 cells. Five out of five mice (100%) without treatment showed stable bioluminescent signal indicating the initiation of tumor while only 1 out of 5 (20%) mice showed tumor initiation in TSA treated group.

To demonstrate the anti-CSC effect of TSA (1 mg/kg, i.p injection, 3 times/week), we utilized mouse xenograft model. We inoculated the mice with 5,000 PANC-1tumor spheres (expressing firefly luciferase) and monitor the tumor initiating ability over time. Five weeks post inoculation, only 1 out of 5 mice (20%) demonstrated bioluminescence, indicating the growth of PANC-1 tumor while all five mice in the vehicle group showed tumor growth (as reflected by the bioluminescence, Fig. 7). This data strongly supported our view that TSA functions as a potential agent for suppressing.

## Discussion

Despite the significant progress made in anticancer therapy, the aggressive PDAC ranking as the fourth leading cause of cancer-related death in the United States, with a 5-year survival rate of 3% and a 0.3% annual increase in death rates, remains a lethal medical challenge^18^, especially as patients continue to be plagued with treatment failure, resistance to therapy and increased potential for locally advanced and metastatic disease, which are probably associated with the presence of the small subset of PDAC cells referred herein to as CSCs with enhanced aggressive phenotype, and which facilitate tumor growth and progression^14^. Thus, necessitating the discovery and developing of therapeutic strategies that effectively target and eliminate these CSCs in PDAC.

In the present study, we provide evidence that, (i) TSA and SAHA, in a HDACs 1, 7 and 8-mediated manner, suppress the viability of human PDAC cells by targeting the CSCs through the modulation of Oct-4, Sox-2 and Nanog expression levels, (ii) TSA induces the accumulation of acetylated histones H3 and H4 in PDAC cells and enhances the epigenetic activity of SAHA, and (iii) the anti-CSCs activity of Gemcitabine is enhanced in the presence of TSA or SAHA^19^.

While HDACs of various classes are dysregulated in PDACs, we demonstrated in this study that the preferential overexpression of HDAC1, HDAC7 and HDAC8 is statistically significant and bear poor prognostic implications in PDACs (Fig. 1). This is consistent with the results from the class I HDACs expression profile of human cancer tissues which revealed an 85% and 90% immunoreactivity for HDAC1 and HDAC8, respectively, as well as studies demonstrating a positive correlation between HDAC1 expression and that of the metastasis-associated protein 1 (MTA1) and hypoxia-inducing factor 1-alpha (HIF-1α) in pancreatic cancer, with the regulation of HIF-1α expression by the HDAC1/MTA1 sub-units of the nucleosome remodeling deacetylase (NuRD) complex, and subsequent association of the HDACs with poor prognosis in PDAC patients (reviewed in^20^). Additionally, HDAC7 has also been implicated as a critical negative regulator of thymocyte activity, which facilitates the down-regulation of the nerve growth factor IB, (Nur77) gene, which is associated with antiproliferative and apoptosis-enhancing activities in PDAC^21^.

We showed the apoptosis mediated anticancer activity of the hydroxamic acids, SAHA and TSAin primary and metastatic PDAC cells (Fig. 2), demonstrating while SAHA and TSA both comprise a hydroxamic-acid-based metal-binding domain that coordinates the catalytic Zn2 + in the HDAC active site, TSA, which harbors a 5-membered carbon-based linker that mimics the C’ functional group of lysine, and a hydrophobic motif that interacts with the periphery of the HDAC binding pocket, exhibited a better therapeutic efficiency over SAHA which has a 6-membered carbon-based linker that mimics the C-terminal functional group of lysine^6^.

We also demonstrated a relationship between the TSA- or SAHA- induced downregulation of HDACs 1, 7 and 8, and the repression of EMT in PDAC, as shown by concurrent upregulation of E-cadherin with suppressed HDAC1, HDAC7, HDAC8 and vimentin expressions (Fig. 3). Since the hallmark of EMT is the loss of epithelial surface marker expression, most notably E-cadherin, and the gain of mesenchymal markers such as vimentin and N-cadherin, we believe our finding highlights the efficacy of the hydroxamic acids in targeting the critical HDAC-mediated tumorigenesis and tumor progression by deregulating PDAC-relevant signaling bio-events such as the EMT, known to be associated with the CSCs phenotypes, including resistance to therapy and metastasis^22,23^. The process of EMT is activated by several signaling pathways, including the reversible epigenetic modifications such as SUMOylation, phosphorylation, methylation, and acetylation, which are critical in the regulation of gene expression by modulating the transcriptional machinery by reshaping the anatomy of chromatin residues at specific genomic loci^24^. Consistent with the documented SNAI1-repression of E-cadherin through histone deacetylation, and our data demonstrating the concurrent upregulation of E-cadherin and co-repression of HDAC1, HDAC7, HDAC8, vimentin, Oct-4, Sox-2 and Nanog after treatment of the PDAC cells with HDAC inhibitor, TSA or SAHA (Fig. 4), we propose that vimentin by direct interaction with E-cadherin promoter, recruits HDAC1, HDAC7 and HDAC8 and the pluripotency transcription factors Oct-4, Sox-2 and Nanog to the E-cadherin (*CDH1)* promoter site, thus silencing CDH1 expression by deacetylation of the histones H3 and H4. Additionally, TSA or SAHA treatment was shown to significantly reduce the tumor sphere formation ability of the PDAC cells. This is in concordance with published findings demonstrating the alteration of CSC-like phenotype in head and neck squamous cell carcinoma treated with the HDAC inhibitors, TSA and SAHA^25^.

Furthermore, we showed that treatment with the hydroxamic acid, TSA, induced the accumulation of accumulation of acetylated histones H3 and H4, significantly suppress the expression of DNMT3B and LSD1, as well as enhance the ability of SAHA to do same (Fig. 5), which is particularly important in the light of the promise of DNA methylation as a therapeutic target in PDAC. DNMTs, such as DNMT3A and DNMT3B serve as writers of the methyl mark, establishing and maintaining the patterns of DNA methylation under diverse circumstances. During tumor formation, aberrant DNA methylation facilitate acquisition of the malignant phenotype, such as demonstrated in PDAC, where DNA methylation is an established tumor suppressor -inactivating mechanism^26^.Our findings in Fig. 5 is also consistent with those of Qin *et al*.^27^, where the genetic silencing of LSD1 repressed the PDAC cell proliferation and tumorigenicity, as well as is therapeutically-relevant especially as LSD1 has been shown to be significantly upregulated in PDAC patient samples, and positively correlated with the overall survival of PDAC patients.

Finally, we demonstrated that TSA enhances the sensitivity of PDACs to Gemcitabine, and that the anti-CSCs activity of Gemcitabine is enhanced in the presence of SAHA or TSA, howbeit, more significant in the presence of TSA (Fig. 6). In support, our *in vivo* study demonstrated TSA treatment significantly suppressed the tumor initiating ability of CSCs derived from PANC-1 (Fig. 7). This is clinically relevant since the most often hypoxic PDACs is a probable CSCs - enriched and -driven pathology, therefore the preferential targeting and elimination of these CSCs which have been implicated in resistance to conventional anticancer therapy, metastasis and recurrence, by TSA or SAHA alone or in combination with Gemcitabine, constitute another step towards the development of a more efficient anti-PDAC therapy in the clinic.

**Figure 8:**
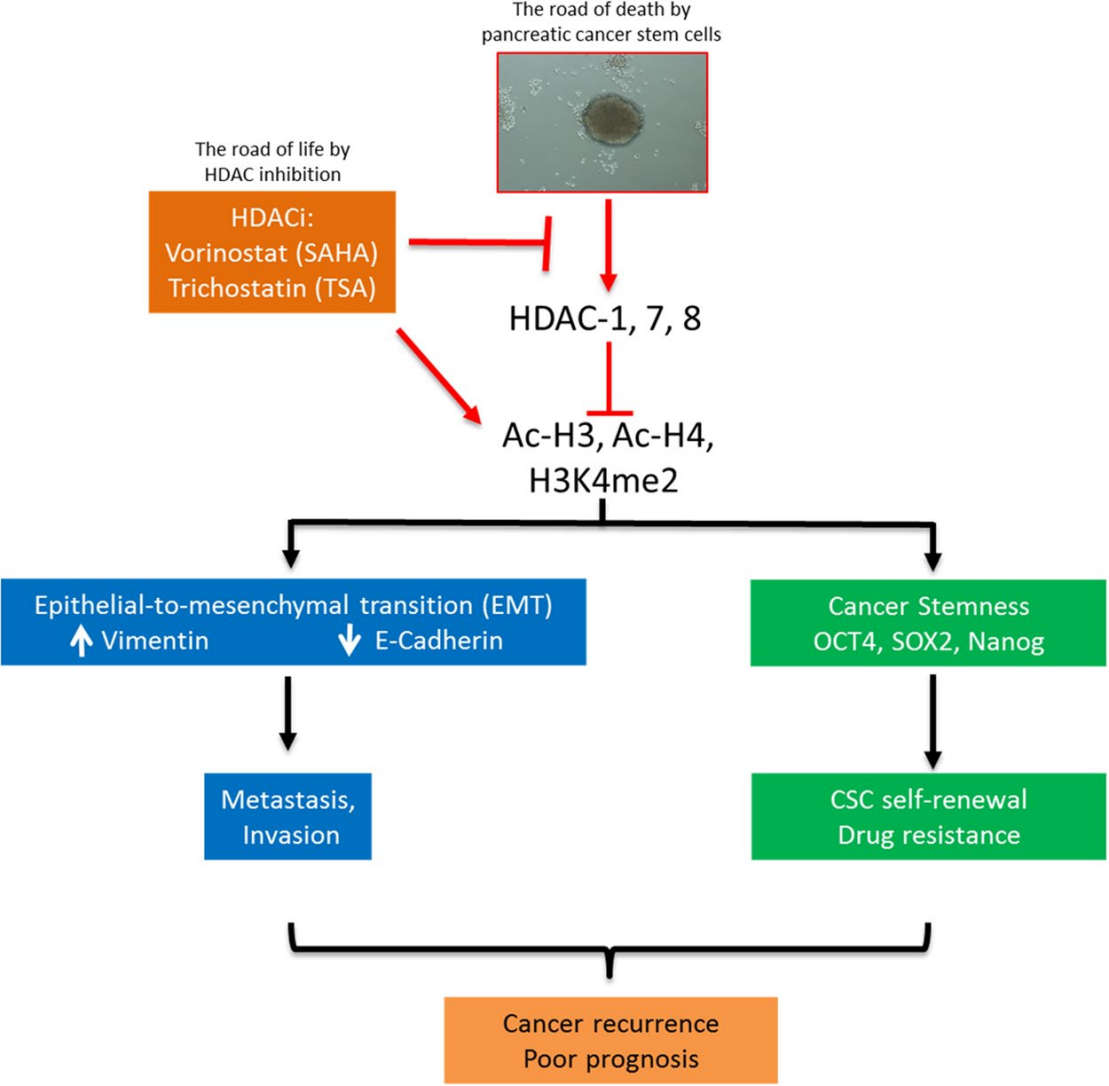
Pictorial Abstract. A schematic summary showing SAHA or TSA inhibits the HDACs 1, 7 and 8 expression/activity by enhancing the cellular accumulation of acetylated histones H3 and H4, as well as methylated lysine 4 tail of histone H3, which subsequently lead to a disbalance in EMT factors in favor of the benign epithelial phenotype, inhibition of cancer metastasis and/or invasion, loss of cancer stemness, increased sensitivity to chemotherapy and better prognosis.

In conclusion, as we depicted in our schematic summary (Fig. 8), we have provided evidence that TSA induces cell death in aggressive human pancreatic cancer cells through the negative modulation of HDACs 1, 7 and 8, as well as the pluripotency transcription factors Oct-4, Sox-2 and Nanog to increase sensitivity of PDAC cells to SAHA or Gemcitabine. In essence, we showed that TSA inhibits the HDACs 1, 7 and 8 expression/activity by enhancing the cellular accumulation of acetylated histones H3 and H4, as well as methylated lysine 4 tail of histone H3, which subsequently lead to a dis-balance in EMT factors in favor of the benign epithelial phenotype, inhibition of cancer metastasis and/or invasion, loss of cancer stemness, increased sensitivity to chemotherapy and better prognosis.

## Materials and Methods

### Drugs and reagents

Trichostatin A (TSA, ≥98% HPLC, T8552SIGMA, Sigma-Aldrich, CA, USA), Vorinostat (SAHA, ≥98% HPLC, SML0061SIGMA, Sigma-Aldrich, CA, USA) and Gemcitabine (≥98% HPLC, G6423SIGMA, Sigma-Aldrich, CA, USA) were in suspended in DMSO, prepared at a stock concentration of 1 mg/ml and stored at −20 °C.

### Cell lines and cell culture

The human pancreatic cancer cell lines, PANC-1 and MiaPaCa-2 was purchased from the American Type Culture Collection (ATCC, Manassas, VA, USA) and were cultured in Dulbecco’s Modified Eagle’s Medium (DMEM, Gibco, Carlsbad, CA, USA) supplemented with 10% fetal bovine serum (FBS), 2 mM L-glutamine 100 U/ml penicillin and 100 g/ml streptomycin (Thermo Fisher Scientific, Inc. Waltham, Ma, USA) in a 5% CO_2_ humidified atmosphere at 37 °C. Cultured cells were passaged at 98% confluence or media changed every 72 h.

### Sulforhodamine B colorimetric cell viability assay

1 × 10^5^ cells/mL PANC-1 or MiaPaCa-2 cells were plated per well containing 200 μl medium in 96-well culture plates overnight, then the cells were treated with different concentrations of TSA, SAHA or Gemcitabine for 48 h for the single drug therapy, while for the combination treatment, cells were treated with TSA for 24 h before addition of SAHA or Gemcitabine, and incubation for another 24 h. Cell viability was examined at specified concentrations of named drugs. After 48 h incubation, cell viability was estimated using the sulforhodamine B assay as previously described^12^. Each experiment was performed at least twice in triplicate, and results are expressed as the mean ± SD.

### Western blot analysis

20 μg of total cell lysates were subjected to a 10% polyacrylamide sodium dodecyl sulfate - polyacrylamide gel electrophoresis (SDS-PAGE) and blots were transferred onto polyvinylidene difluoride (PVDF) membranes. The membranes with the blots were then incubated with 5% non-fat milk in PBS with Tween-20 for 1 h to prevent non-specific binding before being incubated overnight at 4 °C in specific primary antibodies against HDAC1, HDAC7, HDAC8, E-cadherin, Vimentin, Ac-H3, Ac-H4, H3K4me2, H3K9me2 and Histone H3 (Santa Cruz Biotechnology, CA, USA) followed by incubation in peroxidase - conjugated secondary antibody at room temperature for 1 h, washed with PBST three times, then the protein signals were observed using the UVP BioSpectrum system (Analytic Jena Company).

### Bioinformatics Analysis

HDAC gene expression data in the PDAC and adjacent non-tumor tissue were accessed on the public functional genomics data repository, Gene Expression Omnibus (GEO) dataset browser using the series accession number GSE28735.

### Quantitative Real-Time PCR

The RNA expression levels in the treated and untreated control PDAC cells were measured using a RT-qPCR system. After extraction of total RNA using TRIzol reagent (Invitrogen, Thermo Fisher Scientific Inc., Carlsbad, CA, USA), 1 μg of the total RNA was reverse-transcribed using the QuantiTect Reverse Transcription Kit (Qiagen, Germantown, MD, USA). The amplified mRNA level of each specific HDAC was normalized to that of GAPDH. All procedure was performed following manufacturers’ instruction.

### Tumor sphere formation assay

Tumorspheres were generated by plating 1 × 10^4^ treated or untreated control PANC-1 or MIAPaCa-2 cells per well in ultra-low adhesion 6-well plates, containing 2 mL warm StemXVivo serum-free tumorsphere media (CCM012, R&D Systems, Minneapolis, MN, USA) supplemented with 2 U/mL heparin (Sigma) and 0.5 g/mL hydrocortisone (Sigma) following the manufacturer’s protocol, and cells incubated in 5% CO_2_ incubator, at 37 °C for 7–10 days. Tumor spheres were then observed under microscope and those larger than 50 microns counted.

### Annexin V/Propidium Iodide Apoptosis Assay

Assessment of cell death and quantification of apoptotic cells was performed as previously described^13^. Briefly, after harvesting cells, centrifuging and final resuspension in 100 μL 1× Annexin V binding buffer, Annexin V was added according to manufacturer’s instruction, then tubes incubated in the dark room for 15 min, at room temperature. Additional 100 μL of 1× Annexin V binding buffer was added to each reaction tube to obtain 200 μL in each tube, and 4 μL of PI (Sigma) diluted 1:10 in 1× Annexin V binding buffer, then tubes were incubated for 15 min at room temperature in dark room again. Cell were then washed in 500 μL 1× Annexin V binding buffer, centrifuged, supernatant discarded, and cells resuspended in 500 μL 1× Annexin V binding buffer and fixed in 500 μL 2% formaldehyde solution on ice for 10 min. After washing the labeled cells in 1 mL 1× PBS, centrifuging and resuspension, 16 μL of 1:100 diluted RNase A (Sigma) was added to give a final concentration of 50 μg/mL before finally incubating for 15 min at 37 °C and Annexin/PI staining analyses performed.

### Measurement of DNA synthesis

Cells were seeded at a density of 10,000/ml into 96-well plates and incubated with medium- alone or the SAHA or TSA in different concentrations. DNA synthesis was determined by bromodeoxyuridine (BrdU) incorporation, using the Cell Proliferation ELISA, which is based on incorporation of BrdU into newly-synthesized DNA and on antibody-mediated detection of incorporated BrdU, as recommended by the manufacturer.

### *In vivo* evaluation of TSA’s effects on suppressing tumor initiating ability

PANC-1 tumor spheres were generated as described in the main text. Immune compromised NOD/SCID mice were purchased from Biocytogen (Beijing, China). In brief, luciferase-expressing PANC-1 cells were cultured under serum-deprived conditions to generate tumor spheres. After harvesting PANC-1 spheres, spheres were disassociated by the treatment of trypsin (0.025% final concentration). Disassociated PANC-1 sphere cells (5,000) were then mixed with matrigel and injected subcutaneously into the right flank of NOD/SCID mice. The TSA (1 mg/kg/mouse, 3 times/week) treatment was initiated one-week post injection. The tumor initiation process was monitored using the Xenogen IVIS-200 system. Total proteins were extracted and used for immunoblotting. All animal experiments were approved by the Committee of Laboratory Animal Experimentation of Zhejiang University Laboratory Animal Center.

### Statistical Analyses

SPSS v.18.0 for Windows software (SPSS Inc. Chicago, IL, USA) was used for statistical analysis. All data are expressed as mean ± SEM of experiments performed independently at least twice in triplicate. One-way ANOVA and student’s t- test were used to determine the statistical differences between treatment groups. A p value < 0.05 was considered statistically significant.

## Electronic supplementary material

Supplementary Information
