# Supplementary material for: Depletion of HDAC1, 7 and 8 by Histone Deacetylase Inhibition Confers Elimination of Pancreatic Cancer Stem Cells in Combination with Gemcitabine
Source: Sci Rep. 2018 Jan 26;8:1621. doi: 10.1038/s41598-018-20004-0 (PMC5786009; doi:10.1038/s41598-018-20004-0)
Supplement: Supplementary file 1 — Supplementary Information [file 41598_2018_20004_MOESM1_ESM.pdf]

SUPPLEMENTARY INFORMATION

Depletion of HDAC1, 7 and 8 by Histone Deacetylase Inhibition Confers Elimination of Pancreatic Cancer Stem Cells in Combination with Gemcitabine

Mao-Hua Cai[1], Xiao-Gang Xu[2], Shi-Li Yan[1], Ze Sun[1], Yin Ying[3], Bai-Kui Wang[2], Yue-Xing Tu[4,*]

[1]Department of General Surgery, Chun'an First People's Hospital (Zhejiang Province People's Hospital Chun'an Branch), Hangzhou 311700, Zhejiang Province, China

[2] Key Laboratory of Molecular Animal Nutrition of Ministry of Education, Institute of Feed Science, College of Animal Sciences, Zhejiang University, Hangzhou 310029, Zhejiang Province, China

[3] Zhejiang Academy of Traditional Chinese Medicine, Hangzhou 310007, Zhejiang Province, China

[4] Department of Critical Care Medicine, Zhejiang Provincial People’s Hospital, People’s Hospital of Hangzhou Medical College, Hangzhou 310014, Zhejiang Province, China

Corresponding author:

Yue-Xing Tu, PhD

Department of Critical Care Medicine, Zhejiang Provincial People’s Hospital, People’s Hospital of Hangzhou Medical College, No. 158 Shangtang Road, Hangzhou 310014, China; Tel: +86-571-85893281, Fax: +86-571-85893281, E-mail: tuyuexing1988@163.com

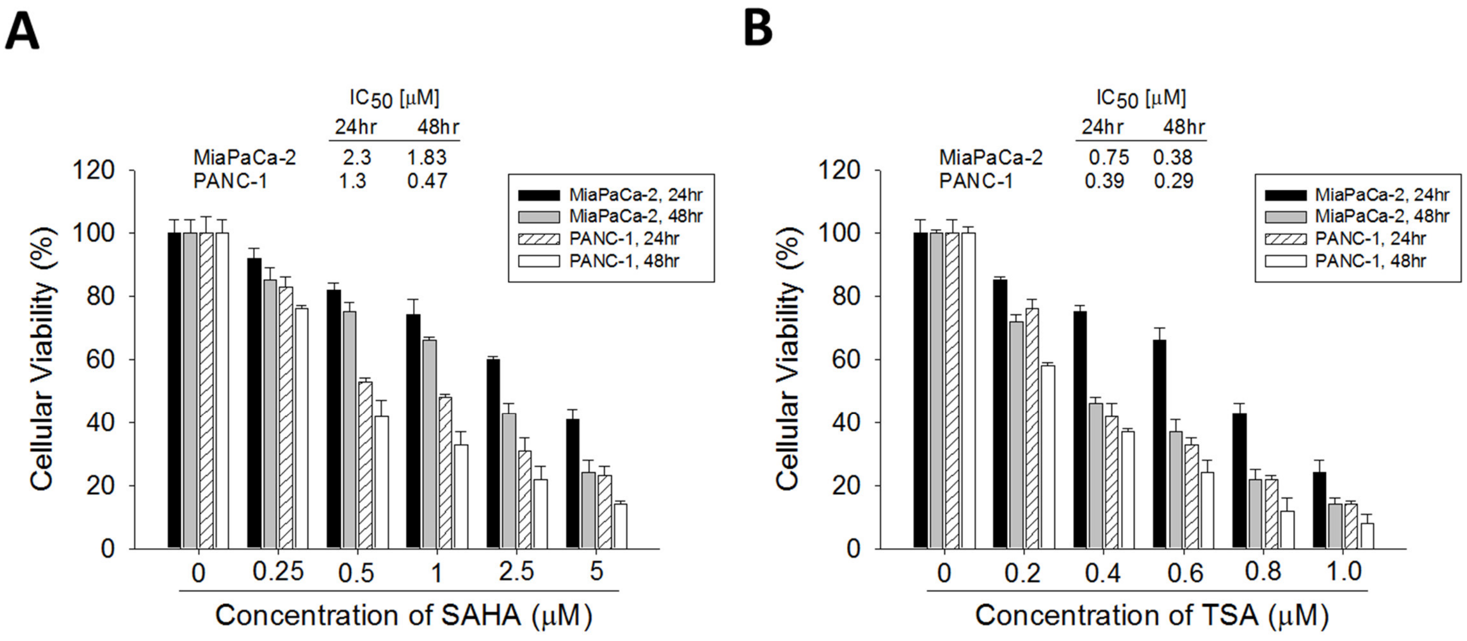

Supplementary Figure S1. Growth inhibitory effects of SAHA and TSA on pancreatic cancer cell lines. (A) PANC-1 and MiaPaCa-2 cells were treated with SAHA (0-5uM) for 24 and 48hrs in 96-well plate. (B) PANC-1 and MiaPaCa-2 cells were treated with TSA (0-1uM) for 24 and 48hrs and cytotoxicity was assessed by SRB assay. The IC50 value was obtained using CalcuSyn software. The results were derived from three independent experiments performed in triplicate.

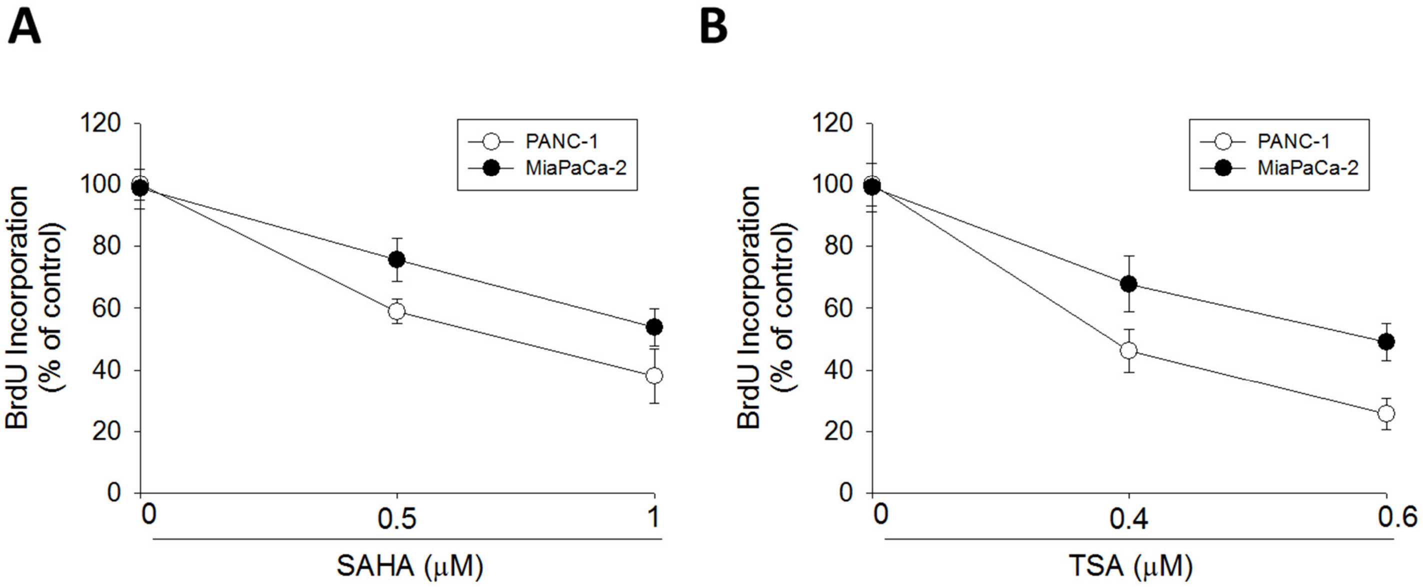

Supplementary Figure S2. SAHA and TSA suppress the growth of pancreatic cancer cells. A and B, Quantification of proliferation of pancreatic cancer cells using the bromodeoxyuridine (BrdU) incorporation assay according to the instruction of the manufacturer. PANC-1 (◯) and MiaPaCa-2(●) cancer cells were treated with SAHA (A) or TSA (B) or vehicle at the indicated concentrations in the presence of 10% fetal bovine serum for 48 hrs. Both SAHA and TSA potently inhibited the proliferation of both cell lines tested.

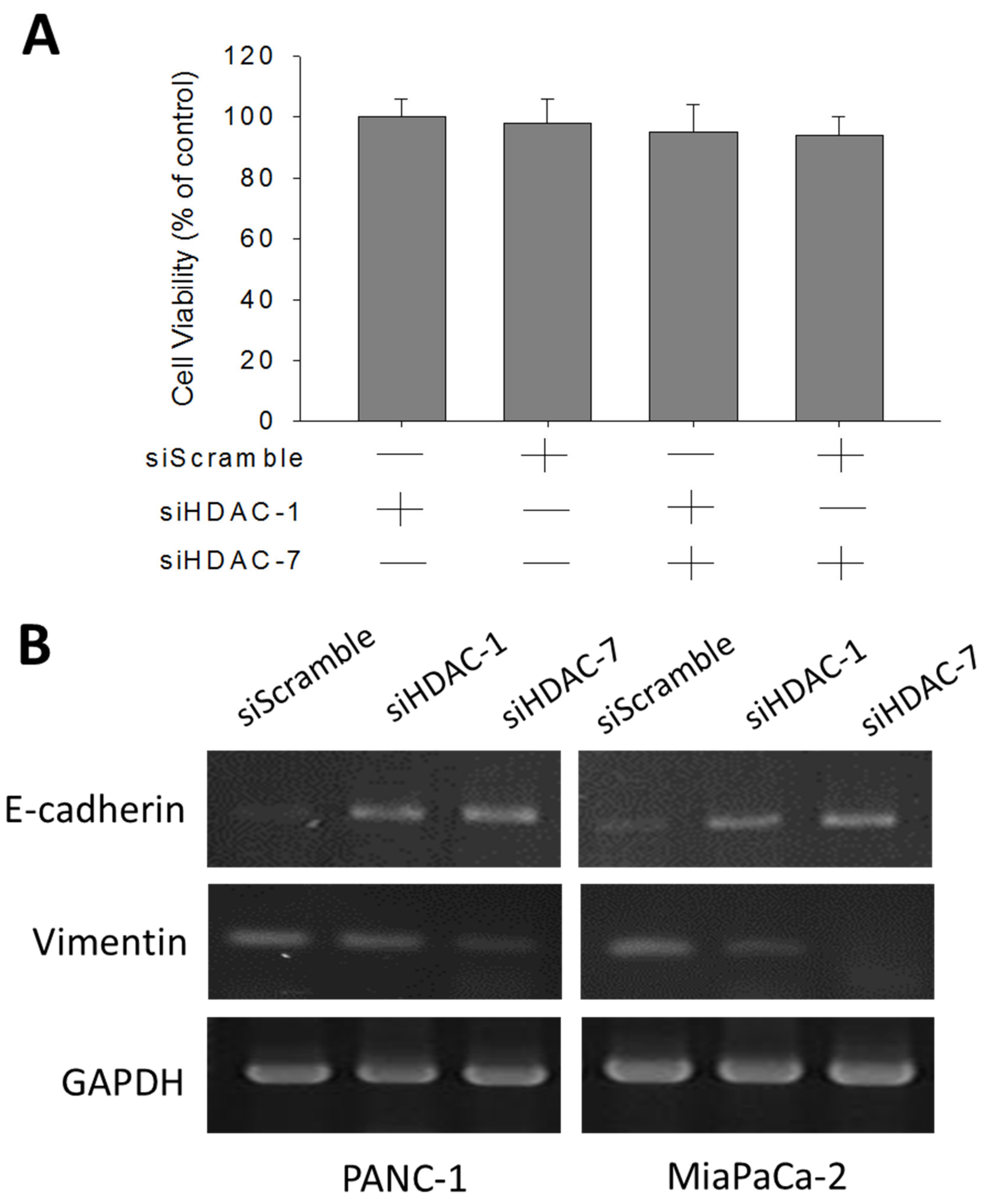

Supplementary Figure S3. Supplementary Figure S3. Depletion of HDAC-1 or HDAC-7 induces the reversion of EMT. (A) The proliferation ability was tested using MTT assay 48 hours after different treatment. Furthermore, no effect on cell proliferation or apoptosis were observed. (B) The upregulation of E-cadherin and the downregulation of vimentin were detected at mRNA in PANC-1 and MiaPaCa-2 cells. These results indicated that HDAC is important for PANC-1 and MiaPaCa-2 cells to maintain mesenchymal characteristics. The results were obtained from three independent experiments.

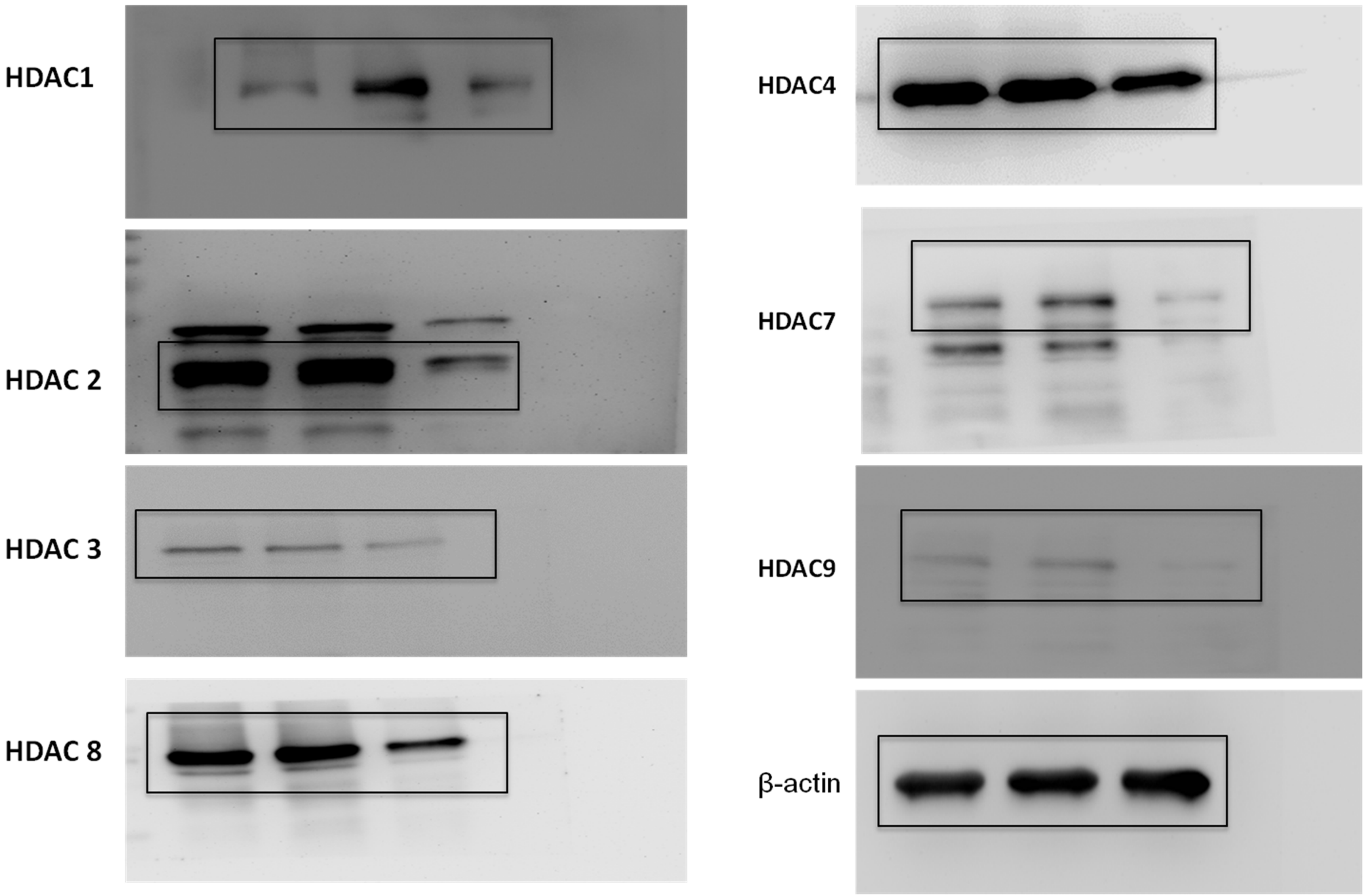

Supplementary Figure S4. Full-size blots of Figure 1D

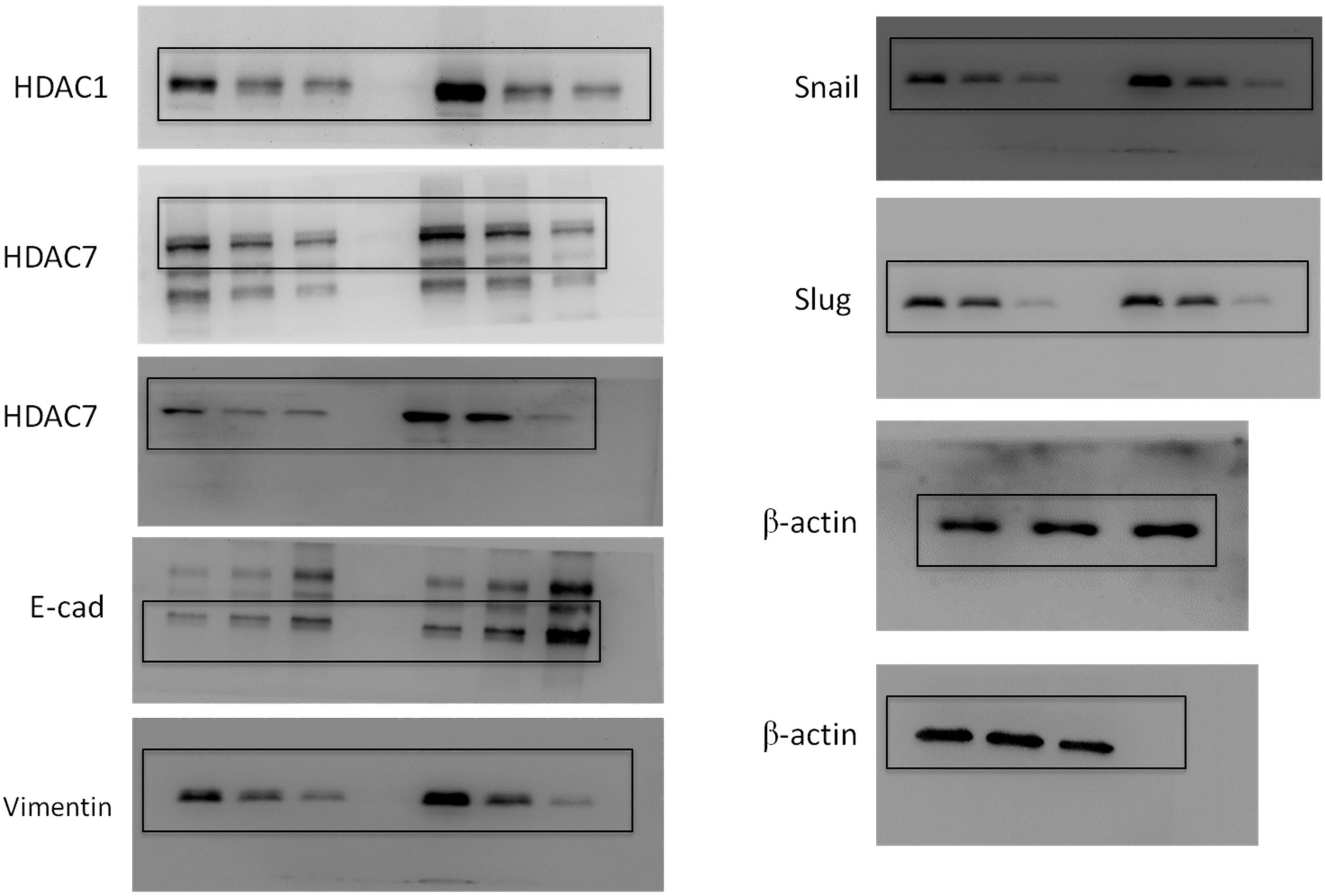

Supplementary Figure S5. Full-size blots of Figure 3C

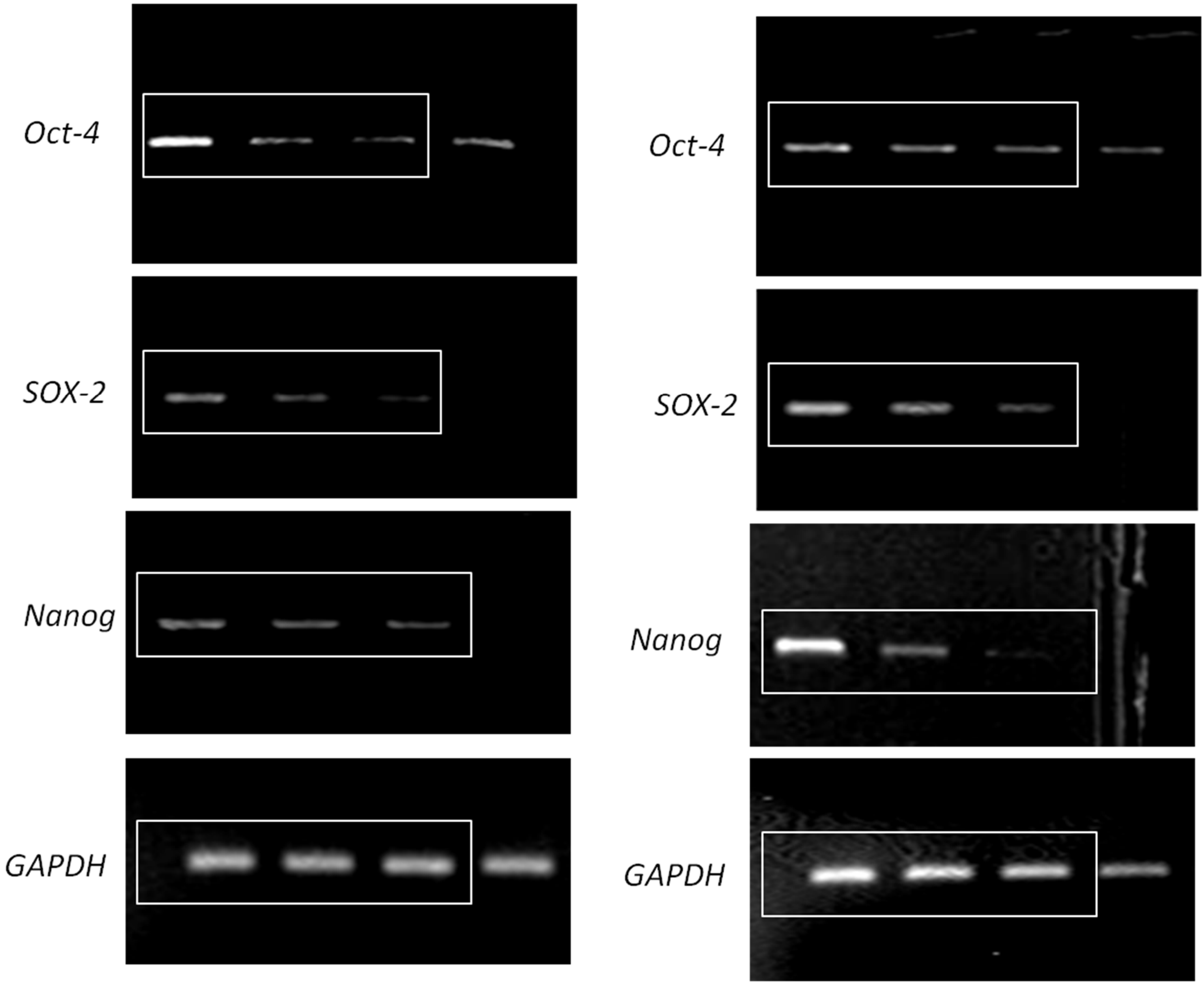

Supplementary Figure S6. Full-size blots of Figure 4A

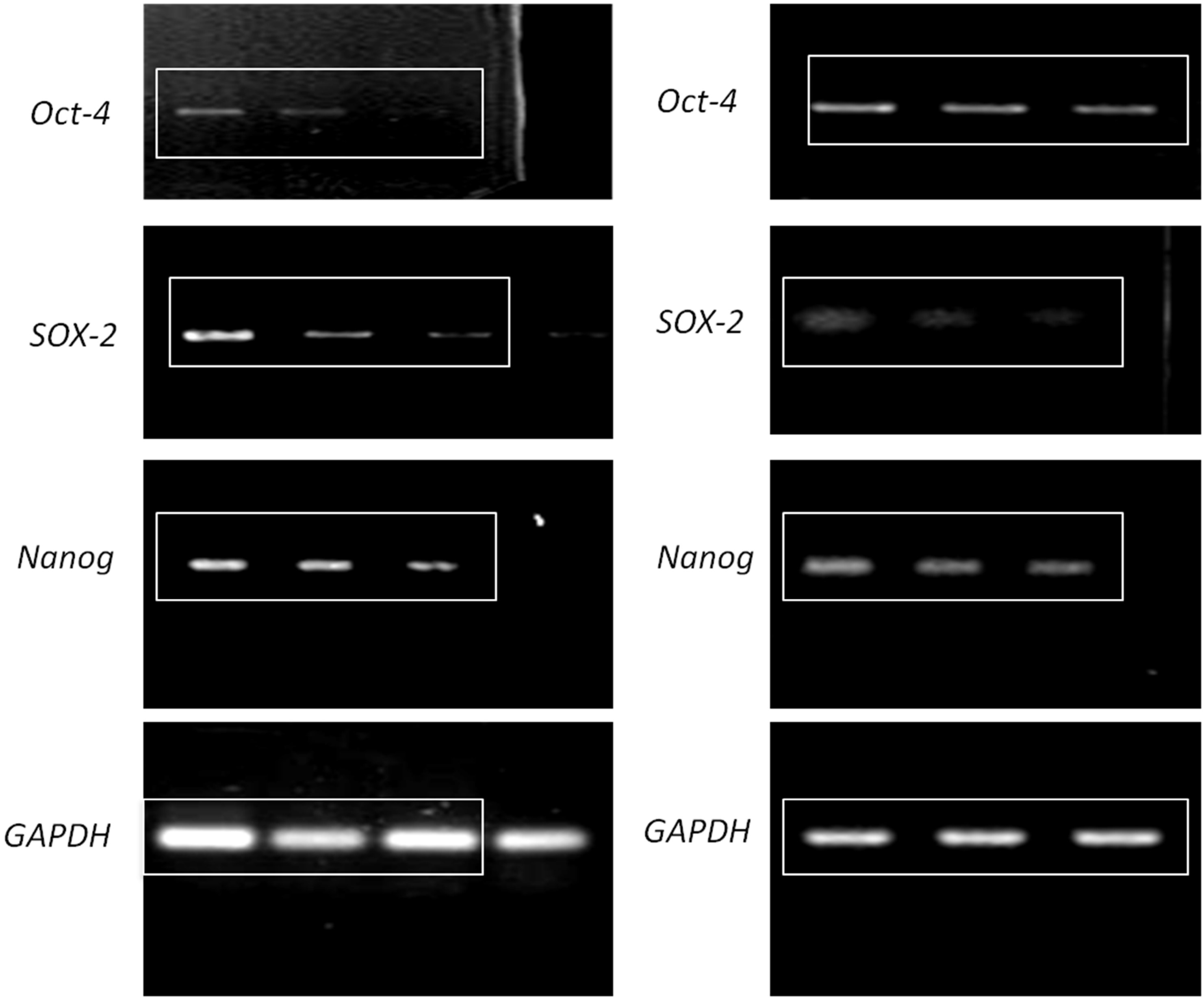

Supplementary Figure S7. Full-size blots of Figure 4B

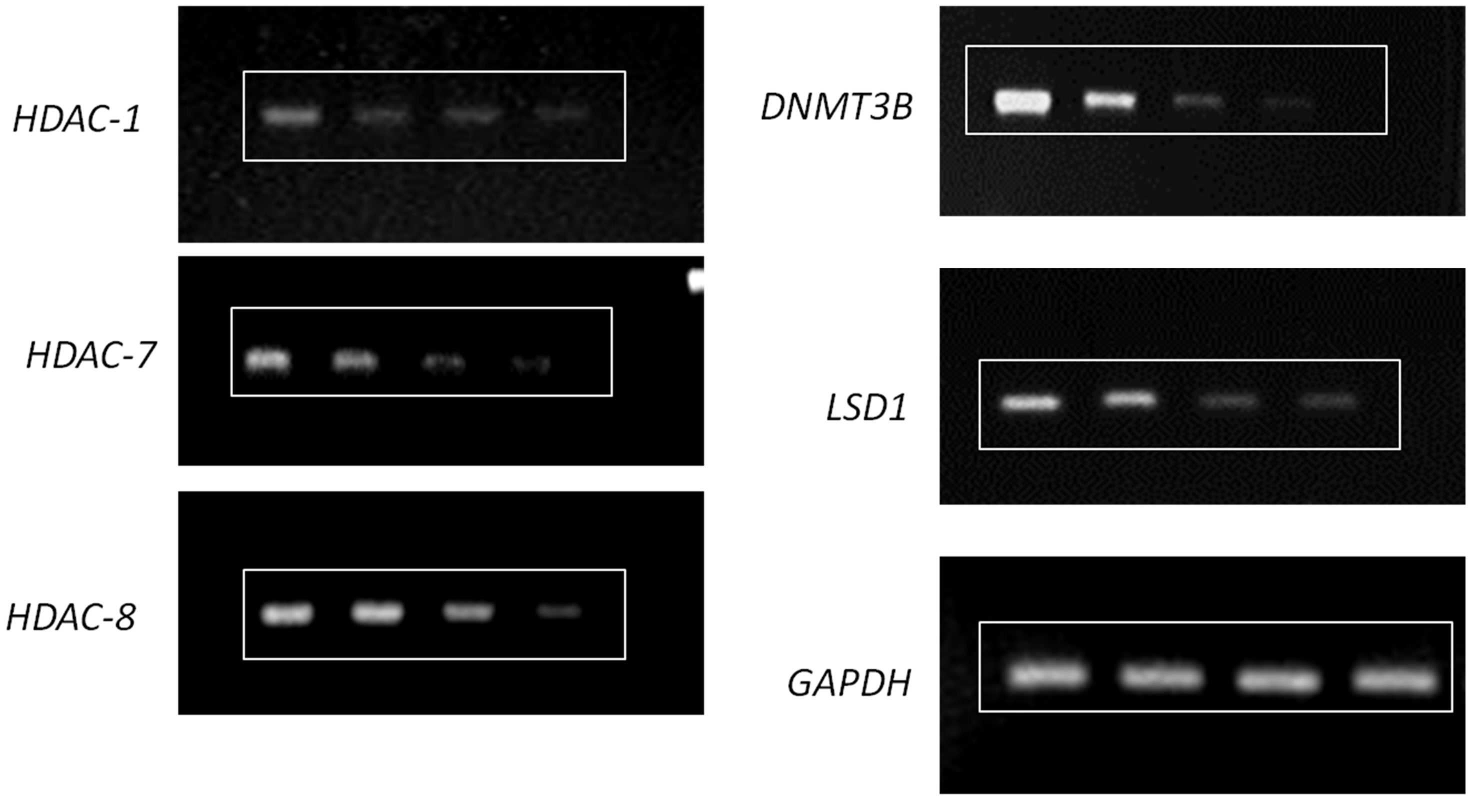

Supplementary Figure S8. Full-size blots of Figure 5A

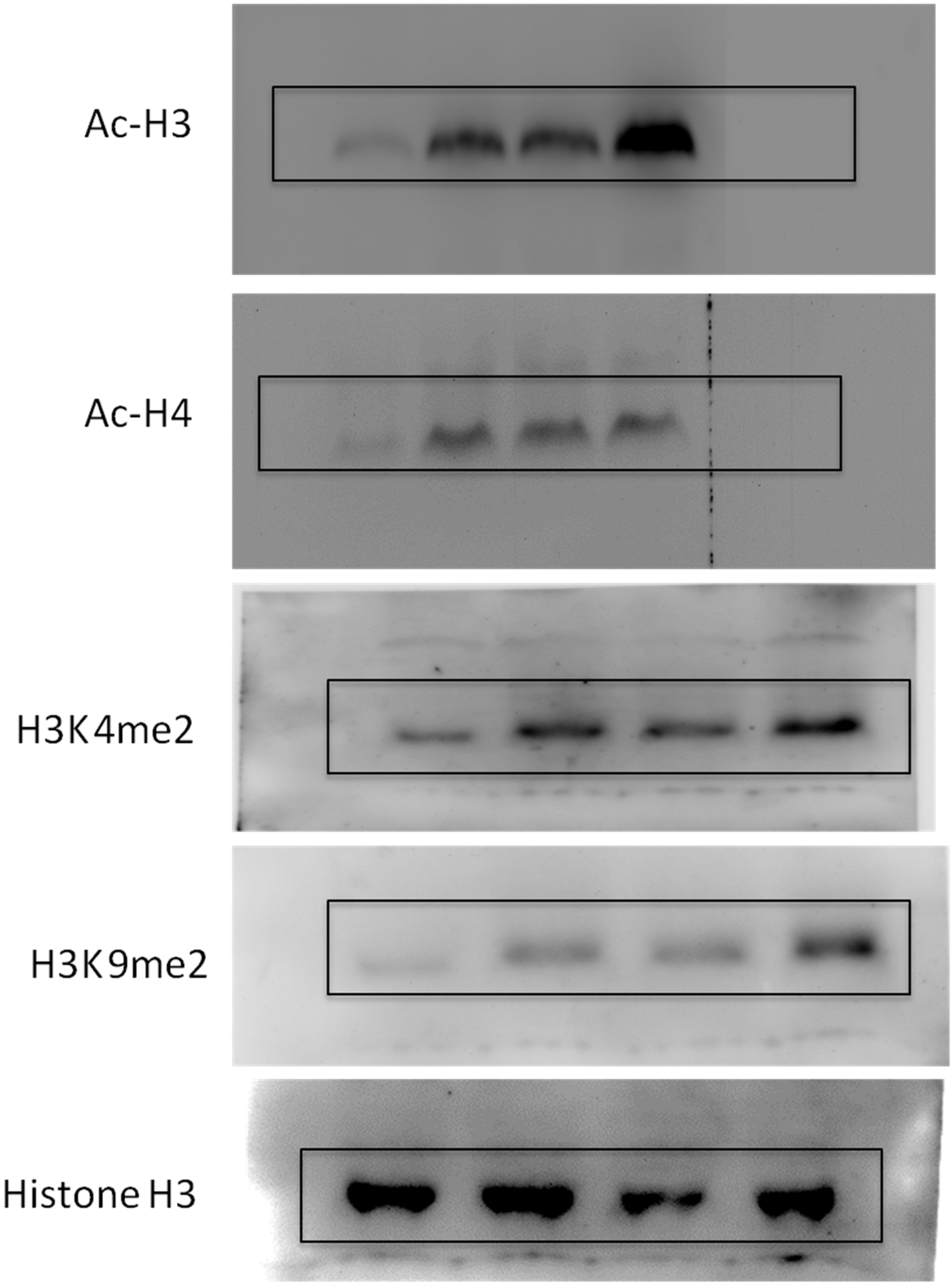

Supplementary Figure S8. Full-size blots of Figure 5B

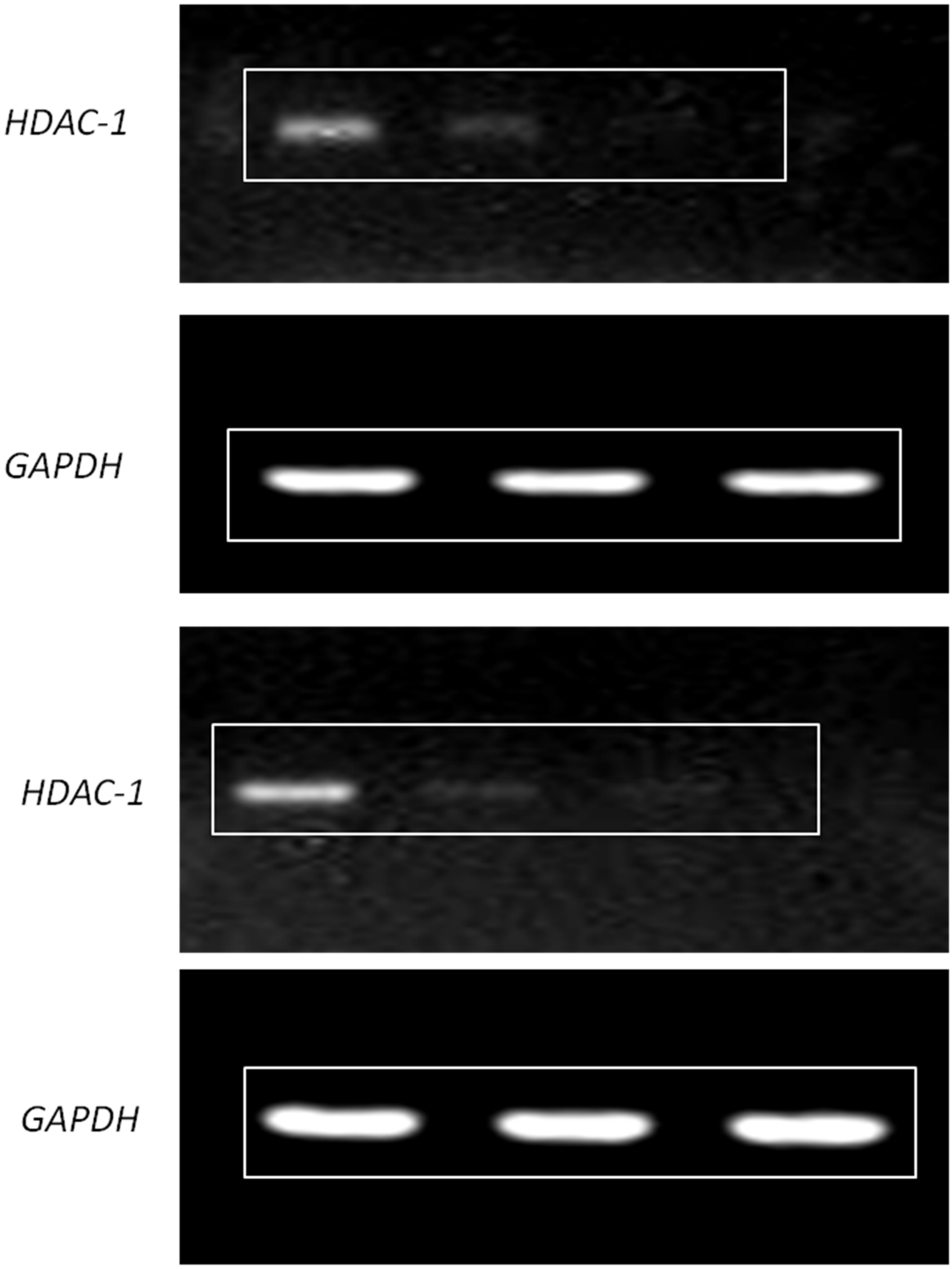

Supplementary Figure S9. Full-size blots of Figure 6A

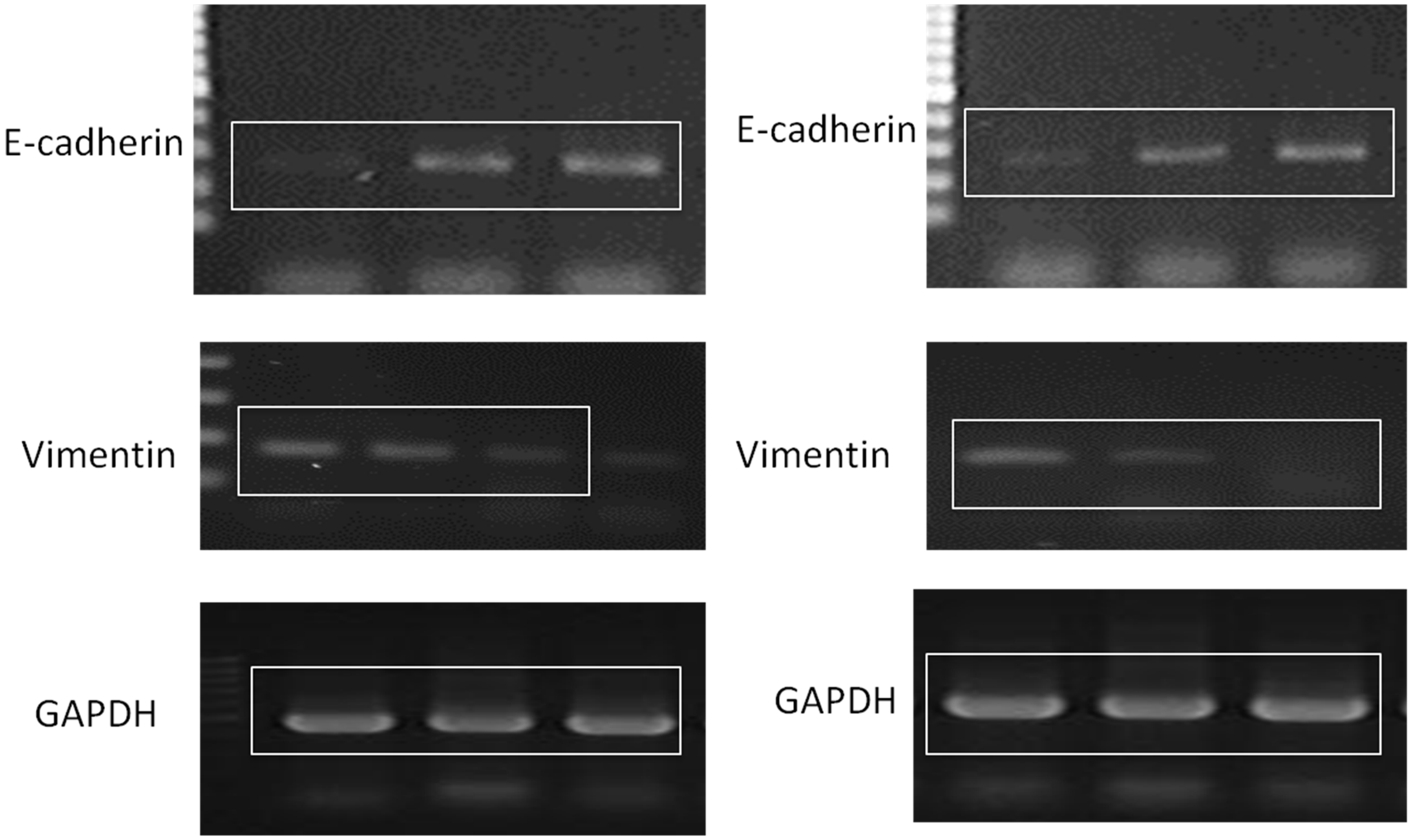

Supplementary Figure S10. Full-size blots of Supplementary Figure S3
